# The l-rhamnose-dependent regulator RhaS and its target promoters from *Escherichia coli* expand the genetic toolkit for regulatable gene expression in the acetic acid bacterium *Gluconobacter oxydans*

**DOI:** 10.3389/fmicb.2022.981767

**Published:** 2022-08-16

**Authors:** Philipp Moritz Fricke, Mandy Lynn Gries, Maurice Mürköster, Marvin Höninger, Jochem Gätgens, Michael Bott, Tino Polen

**Affiliations:** Institute of Bio- and Geosciences, IBG-1: Biotechnology, Forschungszentrum Jülich GmbH, Jülich, Germany

**Keywords:** *Gluconobacter*, rhamnose, regulation, transcription, promoter, activation, repression, acetic acid bacteria

## Abstract

For regulatable target gene expression in the acetic acid bacterium (AAB) *Gluconobacter oxydans* only recently the first plasmids became available. These systems solely enable AraC- and TetR-dependent induction. In this study we showed that the l-rhamnose-dependent regulator RhaS from *Escherichia coli* and its target promoters P*_rhaBAD_*, P*_rhaT_*, and P*_rhaSR_* could also be used in *G. oxydans* for regulatable target gene expression. Interestingly, in contrast to the responsiveness in *E. coli*, in *G. oxydans* RhaS increased the expression from P*_rhaBAD_* in the absence of l-rhamnose and repressed P*_rhaBAD_* in the presence of l-rhamnose. Inserting an additional RhaS binding site directly downstream from the −10 region generating promoter variant P_*rhaBAD*(+RhaS-BS)_ almost doubled the apparent RhaS-dependent promoter strength. Plasmid-based P*_rhaBAD_* and P_*rhaBAD*(+RhaS-BS)_ activity could be reduced up to 90% by RhaS and l-rhamnose, while a genomic copy of P_*rhaBAD*(+RhaS-BS)_ appeared fully repressed. The RhaS-dependent repression was largely tunable by l-rhamnose concentrations between 0% and only 0.3% (w/v). The RhaS-P*_rhaBAD_* and the RhaS-P_*rhaBAD*(+RhaS-BS)_ systems represent the first heterologous repressible expression systems for *G. oxydans*. In contrast to P*_rhaBAD_*, the *E. coli* promoter P*_rhaT_* was almost inactive in the absence of RhaS. In the presence of RhaS, the P*_rhaT_* activity in the absence of l-rhamnose was weak, but could be induced up to 10-fold by addition of l-rhamnose, resulting in a moderate expression level. Therefore, the RhaS-P*_rhaT_* system could be suitable for tunable low-level expression of difficult enzymes or membrane proteins in *G. oxydans*. The insertion of an additional RhaS binding site directly downstream from the *E. coli* P*_rhaT_* −10 region increased the non-induced expression strength and reversed the regulation by RhaS and l-rhamnose from inducible to repressible. The P*_rhaSR_* promoter appeared to be positively auto-regulated by RhaS and this activation was increased by l-rhamnose. In summary, the interplay of the l-rhamnose-binding RhaS transcriptional regulator from *E. coli* with its target promoters P*_rhaBAD_*, P*_rhaT_*, P*_rhaSR_* and variants thereof provide new opportunities for regulatable gene expression in *G. oxydans* and possibly also for simultaneous l-rhamnose-triggered repression and activation of target genes, which is a highly interesting possibility in metabolic engineering approaches requiring redirection of carbon fluxes.

## Introduction

The acetic acid bacterium (AAB) *Gluconobacter oxydans* harbors the beneficial ability of regio- and stereoselective incomplete oxidation of a variety of sugars, sugar alcohols and other substrates in the periplasm by membrane-bound dehydrogenases (mDHs) and release of resulting products into the cultivation medium (Mamlouk and Gullo, 2013; Pappenberger and Hohmann, 2014; Mientus et al., 2017). Therefore, *G. oxydans* is industrially used for oxidative biotransformations of carbohydrates to produce, *e.g*., the tanning lotion additive dihydroxyacetone, the vitamin C precursor l-sorbose, and 6-amino-l-sorbose used for production of the antidiabetic drug miglitol (Ameyama et al., 1981; Saito et al., 1997; Gupta et al., 2001; Tkac et al., 2001; Hekmat et al., 2003; Wang et al., 2016). The industrial versatility of *G. oxydans*, current applications and future perspectives have been reviewed recently (da Silva et al., 2022).

For target gene expression in *G. oxydans*, only constitutive promoters were used in the past due to the lack of a regulatable promoter. For expression, derivatives of the pBBR1MCS plasmid family obtained from the endogenous plasmid pBBR1 from *Bordetella bronchiseptica* were the most successful shuttle and expression vectors used (reviewed in Fricke et al., 2021a). Since pBBR1MCS-2 conferring kanamycine resistance typically results in an abnormal cell morphology of *G. oxydans* in the presence of kanamycin and potentially also in reduced expression performance, pBBR1MCS-5 and the use of gentamicin is advantageous (Fricke et al., 2021b). However, both plasmid backbones recently enabled high functionality of transferred heterologous expression systems for regulatable target gene expression in *G. oxydans* for the first time. Firstly, the l-arabinose-dependent AraC-P*_araBAD_* system from *Escherichia coli* MC4100, which exhibits a better *araC* codon usage in *G. oxydans* than *araC* from *E. coli* MG1655, was tunable and inducible up to 480-fold (Fricke et al., 2020). Interestingly, in *G. oxydans* the AraC target promoter P*_araBAD_* from *E. coli* was not active in the absence of AraC. This indicated that P*_araBAD_* alone is not recognized by the *G. oxydans* RNA polymerase. Therefore, the typical repression of P*_araBAD_* by AraC in the absence of the inducer l-arabinose was not required to ensure non-induced tightness of P*_araBAD_* in *G. oxydans*. Secondly, the TetR-P*_tet_* system in its native divergent organization as present in the *E. coli* transposon Tn*10* exhibited extremely low basal expression in *G. oxydans* and achieved more than 3,500-fold induction according to reporter assays using the fluorescence protein mNeonGreen (Fricke et al., 2021b). In contrast to P*_araBAD_* and AraC, P*_tet_* highly required the repression by its regulator TetR for tightness of the system, otherwise the expression from P*_tet_* was very strong in *G. oxydans* without TetR. Moreover, in cases where the native divergent organization *tetR*-P*_tetR_*-P*_tet_*-gene-of-interest is leaky, modifying the genetic organization that the target gene and *tetR* expression both are under control of P*_tet_* and therefore expressed as an operon and auto-regulated by TetR, can improve the non-induced tightness and the resulting inducibility of P*_tet_* in *G. oxydans* (Bertucci et al., 2022).

In this study, to expand the still very limited genetic toolbox for regulatable target gene expression in *G. oxydans* we chose to test the l-rhamnose-dependent RhaSR system from *E. coli* (Baldoma et al., 1990; Egan and Schleif, 1993, 1994; Vía et al., 1996; Bhende and Egan, 1999; Wickstrum et al., 2010). Compared to the AraC-, TetR-, and LacI-based systems from *E. coli*, the RhaRS system offers special features that could be particularly interesting and useful for applications in *G. oxydans* or AAB in general (Supplementary Figure S1). Firstly, the system comprises not only one, but two transcriptional regulators, RhaR and RhaS, both responding to l-rhamnose. They are encoded by the *rhaSR* operon and are expressed from the promoter P*_rhaSR_*. In *E. coli*, basal expression from P*_rhaSR_* is positively auto-regulated by RhaR in the presence of l-rhamnose, resulting in increased expression of the *rhaSR* operon and in turn P*_rhaSR_* is negatively auto-regulated by RhaS since RhaS is also able to bind to the RhaR binding site at P*_rhaSR_*, competing with RhaR and blocking *rhaSR* expression. Secondly, the major target promoters of RhaS are P*_rhaBAD_* and P*_rhaT_*. P*_rhaBAD_* drives transcription of the structural *rhaBAD* genes encoding the l-rhamnose catabolic enzymes l-rhamnulose kinase, l-rhamnose isomerase and l-rhamnulose-1-phosphate aldolase. P*_rhaT_* drives transcription of *rhaT* encoding an l-rhamnose transport system. In *E. coli*, RhaS activates transcription from P*_rhaBAD_* and P*_rhaT_* in the presence of l-rhamnose. Furthermore, in *E. coli* the l-rhamnose metabolism is under catabolite repression by glucose, which is overcome by the binding of the cAMP receptor protein (CRP) to consensus recognition sequences found in all three P*_rha_* promoters and interaction of CRP with the RNA polymerase, which depends on the bending of the promoter DNA by RhaS or RhaR. In *G. oxydans* CRP is absent since the predicted CRP gene (GOX0974/GOX_RS06010) was shown to encode an iron–sulfur cluster protein termed GoxR, an FNR-type transcriptional regulator of genes involved in respiration and redox metabolism (Schweikert et al., 2021). Overall, it seemed very interesting to analyze how RhaS, RhaR, and the promoters P*_rhaBAD_*, P*_rhaT_*, and P*_rhaSR_* perform in *G. oxydans* and if they could be useful for regulatable gene expression in this AAB.

We found that in *G. oxydans* the RhaS-dependent regulation of P*_rhaBAD_* surprisingly was reversed compared to *E. coli*. In the absence of l-rhamnose, RhaS increased expression from P*_rhaBAD_* and in the presence of l-rhamnose RhaS repressed P*_rhaBAD_* enabling complete repression of a genomically encoded P*_rhaBAD_* promoter variant, thereby potentially providing a dynamic knock-down system for genes in *G. oxydans*. The effects and properties of the l-rhamnose-binding RhaS regulator and the promoters P*_rhaBAD_*, P*_rhaT_*, and P*_rhaSR_* from *E. coli* exhibit very interesting characteristics in *G. oxydans* and provide new opportunities for regulatable gene expression, both in fundamental research and metabolic engineering approaches.

## Materials and methods

### Bacterial strains, plasmids, media and growth conditions

Bacterial strains and plasmids used in this study and their relevant characteristics are listed in Table 1. *G. oxydans* cells were routinely cultivated in d-mannitol complex medium containing 40 g L^−1^ d-mannitol, 5 g L^−1^ yeast extract, 1 g L^−1^ KH_2_PO_4_, 1 g L^−1^ (NH_4_)_2_SO_4_, and 2.5 g L^−1^ MgSO_4_ × 7 H_2_O at 30°C. The initial pH of the medium was set to 6 by the addition of KOH (5 M stock). Because *G. oxydans* possesses a natural resistance toward cefoxitin, 50 μg ml^−1^ of the antibiotic was routinely added to the medium as a precaution to prevent bacterial contaminations. Stock solutions of cefoxitin (50 mg ml^−1^) and d-mannitol (200 g L^−1^) were sterile-filtered and added to autoclaved medium. Unless stated otherwise, for shake flask cultivations cells from 10 ml overnight pre-cultures were used to inoculate 50 ml d-mannitol medium in 500 ml shaking flasks with three baffles to an initial optical density at 600 nm (OD_600_) of 0.3 (UV-1800, Shimadzu). All shake flasks cultures were grown on a rotary shaker at an agitation speed of 180 rpm. *G. oxydans* cells harboring pBBR1MCS-5-based plasmids were supplemented with 10 μg ml^−1^ gentamicin (Kovach et al., 1994). *Escherichia coli* strains were cultivated at 37°C and 160 rpm in lysogeny broth (LB) medium. Medium of *E. coli* carrying pBBR1MCS-5-based plasmids was supplemented with 10 μg ml^−1^ gentamicin. *Escherichia coli* S17-1 was used as donor strain to transform *G. oxydans* by conjugation (Kiefler et al., 2017). Competent *E. coli* S17-1 were prepared and transformed by CaCl_2_ procedure as described (Hanahan, 1983).

**Table 1:** Strains and plasmids used or constructed in this study.

Strain	Relevant characteristics	Reference/Source
*E. coli* S17-1	Δ*recA*, *endA1*, *hsdR17*, *supE44*, *thi*-1, *tra*^+^	Simon et al. (1983)
*Gluconobacter oxydans* 621H	DSM 2343	DSMZ
*G. oxydans mNG*	Derivative of *G. oxydans* 621H with reporter gene *mNG* under control of P_*rhaBAD*(+RhaS-BS)_ integrated into the intergenic region igr3 (GOX0038/GOX_RS01330–GOX0039/GOX_RS01335)	This work
*G. oxydans mNG* igr2::P_GOX0264_-*rhaS*	Derivative of *G. oxydans mNG* with *rhaS* under control of P_GOX0264_ integrated into igr2 (GOX0028/GOX_RS01280 - GOX0029/GOX_RS01285)	This work
*G. oxydans mNG* igr2::P*_rhaSR_*-*rhaS*	Derivative of *G. oxydans mNG* with *rhaS* under control of P*_rhaSR_* integrated into igr2 (GOX0028/GOX_RS01280–GOX0029/GOX_RS01285)	This work
*G. oxydans mNG* igr1::P_GOX0264_-*rhaS* igr2::*rhaS*	Derivative of *G. oxydans mNG* igr2::P_GOX0264_-*rhaS* with a second copy of *rhaS* under control of P_GOX0264_ integrated into igr1 (GOX0013/GOX_RS01200–GOX0014/GOX_RS01205)	This work
*G. oxydans mNG* igr1::P*_rhaSR_*-*rhaS* igr2::*rhaS*	Derivative of *G. oxydans mNG* igr2::P_GOX0264_-*rhaS* with a second copy of *rhaS* under control of P*_rhaSR_* integrated into igr1 (GOX0013/GOX_RS01200–GOX0014/GOX_RS01205)	This work
**Plasmid**		
pBBR1MCS-5	Derivative of pBBR1MCS; Gm^R^	Kovach et al. (1995)
pBBR1MCS-5-T*_gdhM_*-MCS-T_GOX0028_	Derivative of pBBR1MCS-5 with terminator sequences of GOX0265 (T*_gdhM_*) and GOX0028 (T_GOX0028_) flanking the multiple cloning site	Fricke et al. (2021b)
pBBR1MCS-5-*rhaSR*-P*_rhaSR_*-P*_rhaBAD_*-*mNG*	Derivative of pBBR1MCS-5-T*_gdhM_*-MCS-T_GOX0028_ with DNA fragment *rhaSR*-P*_rhaSR_*-P*_rhaBAD_* from *E. coli* with l-rhamnose-regulated promoter P*_rhaBAD_* controlling expression of the fluorescent reporter gene *mNG*	This work
pBBR1MCS-5-*rhaS*-P*_rhaSR_*-P*_rhaBAD_*-*mNG*	Derivative of pBBR1MCS-5-*rhaSR*-P*_rhaSR_*-P*_rhaBAD_*-*mNG* lacking the regulator gene *rhaR*	This work
pBBR1MCS-5-*rhaR*-P*_rhaSR_*-P*_rhaBAD_*-*mNG*	Derivative of pBBR1MCS-5-*rhaSR*-P*_rhaSR_*-P*_rhaBAD_*-*mNG* lacking the regulator gene *rhaS*	This work
pBBR1MCS-5-P*_rhaSR_*-P*_rhaBAD_*-*mNG*	Derivative of pBBR1MCS-5-*rhaSR*-P*_rhaSR_*-P*_rhaBAD_*-*mNG* lacking the *rhaSR* operon	This work
pBBR1MCS-5-*rhaS*-P_GOX0264_-P*_rhaBAD_*-*mNG*	Derivative of pBBR1MCS-5-*rhaS*-P*_rhaSR_*-P*_rhaBAD_*-*mNG* with *rhaS* constitutively expressed from strong promoter P_GOX0264_	This work
pBBR1MCS-5-*rhaS*-P_GOX0452_-P*_rhaBAD_*-*mNG*	Derivative of pBBR1MCS-5-*rhaS*-P*_rhaSR_*-P*_rhaBAD_*-*mNG* with *rhaS* constitutively expressed from moderate promoter P_GOX0452_	This work
pBBR1MCS-5-*mNG*-P*_rhaSR_*-P_GOX0264_-*rhaS*	Derivative of pBBR1MCS-5-*rhaS*-P*_rhaSR_*-P*_rhaBAD_*-*mNG* with *mNG* expressed from P*_rhaSR_* and, in opposite direction, *rhaS* constitutively expressed from strong promoter P_GOX0264_	This work
pBBR1MCS-5-*mNG*-P*_rhaSR_*-P_GOX0452_-*rhaS*	Derivative of pBBR1MCS-5-*rhaS*-P*_rhaSR_*-P*_rhaBAD_*-*mNG* with *mNG* expressed from P*_rhaSR_* and, in opposite direction, *rhaS* constitutively expressed from moderate promoter P_GOX0452_	This work
pBBR1MCS-5-*mNG*-P*_rhaSR_*	Derivative of pBBR1MCS-5-*mNG*-P*_rhaSR_*-P_GOX0264_-*rhaS* lacking P_GOX0264_-*rhaS*	This work
pBBR1MCS-5-*rhaS*-P*_rhaSR_*-P_*rhaBAD*(+RhaS-BS)_-*mNG*	Derivative of pBBR1MCS-5-*rhaS*-P*_rhaSR_*-P*_rhaBAD_*-*mNG* with an additional copy of the RhaS binding site (+RhaS-BS) in P*_rhaBAD_* directly downstream from the −10 region	This work
pBBR1MCS-5-P*_rhaSR_*-*rhaS*	Derivative of pBBR1MCS-5 to expresses *rhaS* under control of P*_rhaSR_* (pBBR1MCS-5-T*_gdhM_*-P*_rhaSR_*-*rhaS*-T_GOX0028_)	This work
pBBR1MCS-5-*rhaS*-P*_rhaSR_*-P*_rhaT_*-*mNG*	Derivative of pBBR1MCS-5-*rhaS*-P*_rhaSR_*-P*_rhaBAD_*-*mNG* with P*_rhaT_* controlling *mNG* expression	This work
pBBR1MCS-5-P*_rhaT_*-*mNG*	Derivative of pBBR1MCS-5-*rhaS*-P*_rhaSR_*-P*_rhaT_*-*mNG* lacking regulator gene *rhaS*	This work
pBBR1MCS-5-*rhaS*-P_GOX0264_-P*_rhaT_*-*mNG*	Derivative of pBBR1MCS-5-*rhaS*-P*_rhaSR_*-P*_rhaT_*-*mNG* with P_GOX0264_ controlling *rhaS* expression	This work
pBBR1MCS-5-*rhaS*-P_GOX0452_-P*_rhaT_*-*mNG*	Derivative of pBBR1MCS-5-*rhaS*-P*_rhaSR_*-P*_rhaT_*-*mNG* with P_GOX0452_ controlling *rhaS* expression	This work
pBBR1MCS-5-*rhaS*-P_GOX0264_-P_*rhaT*(−10-RhaS-BS)_-*mNG*	Derivative of pBBR1MCS-5-*rhaS*-P_GOX0264_-P*_rhaT_*-*mNG* with additional RhaS binding site in P*_rhaT_* directly downstream from the −10 region	This work
pBBR1MCS-5-*rhaS*-P_GOX0452_-P_*rhaT*(−10-RhaS-BS)_-*mNG*	Derivative of pBBR1MCS-5-*rhaS*-P_GOX0452_-P*_rhaT_*-*mNG* with additional RhaS binding site in P*_rhaT_* directly downstream from the −10 region	This work
pBBR1MCS-5-*rhaS*-P_GOX0264_-P_*rhaT*(TSS-RhaS-BS)_-*mNG*	Derivative of pBBR1MCS-5-*rhaS*-P_GOX0264_-P*_rhaT_*-*mNG* with additional RhaS binding site in P*_rhaT_* directly downstream from the *E. coli* transcriptional start	This work
pBBR1MCS-5-*rhaS*-P_GOX0452_-P_*rhaT*(TSS-RhaS-BS)_-*mNG*	Derivative of pBBR1MCS-5-*rhaS*-P_GOX0452_-P*_rhaT_*-*mNG* with additional RhaS binding site in P*_rhaT_* directly downstream from the *E. coli* transcriptional start	This work
pKOS6b	Derivative of pAJ63a, *upp* removed, *codBA* integrated, Km^R^, confers 5-fluorocytosine sensitivity (FC^S^)	Kostner et al. (2013)
pKOS6b-igr3::*mNG*	Derivative of pKOS6b for genomic integration of P_*rhaBAD*(+RhaS-BS)_-RBS-*mNG*-T_BBa_B1002_-T_GOX0028_ into igr3 (GOX0038/GOX_RS01330–GOX0039/GOX_RS01335)	This work
pKOS6b-igr2::P_GOX0264_-*rhaS*	Derivative of pKOS6b for genomic integration of P_GOX0264_-*rhaS*-T*_gdhM_* into igr2 (GOX0028/GOX_RS01280–GOX0029/GOX_RS01285)	This work
pKOS6b-igr2::P*_rhaSR_*-*rhaS*	Derivative of pKOS6b for genomic integration of P*_rhaSR_*-*rhaS*-T*_gdhM_* into igr2 (GOX0028/GOX_RS01280–GOX0029/GOX_RS01285)	This work
pKOS6b-igr1::P_GOX0264_-*rhaS*	Derivative of pKOS6b for genomic integration of P_GOX0264_-*rhaS*-T*_gdhM_* into igr1 (GOX0013/GOX_RS01200–GOX0014/GOX_RS01205)	This work
pKOS6b-igr1::P*_rhaSR_*-*rhaS*	Derivative of pKOS6b for genomic integration of P*_rhaSR_*-*rhaS*-T*_gdhM_* into igr1 (GOX0013/GOX_RS01200–GOX0014/GOX_RS01205)	This work

### Recombinant DNA work

All DNA oligonucleotides used in this study were obtained from Eurofins Genomics and are listed in Supplementary Table S1. All enzymes required for recombinant DNA work were purchased from Thermo Scientific. Polymerase chain reactions (PCR) used for DNA manipulation and plasmid verification followed standard protocols as described (Sambrook et al., 1989). For amplification of DNA fragments Q5 DNA polymerases was utilized as recommended by the manufacturer (New England Biolabs). All reporter plasmids were constructed in a one-step isothermal Gibson assembly (50°C, 1 h) by integrating amplified DNA fragments into the restricted broad-host vector derivative pBBR1MCS-5-T*_gdhM_*-MCS-T_GOX0028_ (Gibson et al., 2009). All DNA modifications to create the desired plasmids were conducted in *E. coli* S17-1. For plasmid isolation a QIAprep spin miniprep kit (Qiagen) was used according to the manufacturer’s protocol. The correctness of the plasmid inserts was checked by DNA sequencing (Eurofins MWG).

### Construction of plasmids

In this study, all plasmids were constructed using the vector pBBR1MCS-5-T*_gdhM_*-MCS-T_GOX0028_ that we created previously for the TetR-P*_tet_* system (Fricke et al., 2021b). The terminator sequences of GOX0265 (T*_gdhM_*) and GOX0028 (T_GOX0028_) flank the multiple cloning site (MCS) to reduce potential interferences caused by genetic elements on the plasmid backbone. Unless stated otherwise, pBBR1MCS-5-T*_gdhM_*-MCS-T_GOX0028_ was restricted for insert integration with the restriction endonucleases *Xba*I and *Eco*RI. Furthermore, in all constructs using the promoters P*_rhaBAD_*, P*_rhaSR_*, P*_rhaT_*, P_GOX0264_, or P_GOX0452_ to express the reporter gene *mNeonGreen* (*mNG*), the ribosome binding site (RBS) AGGAGA was placed upstream from *mNG* and downstream from the naturally occurring RBS of the respective promoter region.

For construction of plasmid pBBR1MCS-5-*rhaSR*-P*_rhaSR_*-P*_rhaBAD_*-*mNG*, two DNA fragments were inserted in pBBR1MCS-5-T*_gdhM_*-MCS-T_GOX0028_: DNA fragment with *rhaSR*-P*_rhaSR_*-P*_rhaBAD_*-RBS amplified with the primer pair PF1/PF2 from the genome of *E. coli* LJ110 and DNA fragment with *mNG*-T_BBa_B1002_ amplified with the primer pair PF3/PF4 from pBBR1MCS-5-*araC*-P*_BAD_-mNG* (Fricke et al., 2020). The latter DNA fragment included the terminator BBa_B1002 from the iGEM parts library directly downstream from the reporter gene *mNG*.

The plasmid pBBR1MCS-5-*rhaS*-P*_rhaSR_*-P*_rhaBAD_*-*mNG* lacking *rhaR* was constructed with a DNA fragment amplified with the primer pair PF5/PF4 from pBBR1MCS-5-*rhaSR*-P*_rhaSR_*-P*_rhaBAD_*-*mNG* resulting in fragment *rhaS*-P*_rhaSR_*-P*_rhaBAD_*-RBS-*mNG*-T_BBa_B1002_ and subsequent integration of this fragment into pBBR1MCS-5-T*_gdhM_*-MCS-T_GOX0028_.

The plasmid pBBR1MCS-5-*rhaR*-P*_rhaSR_*-P*_rhaBAD_*-*mNG* lacking *rhaS* was constructed with a DNA fragment containing *rhaR* and a DNA fragment containing P*_rhaSR_*-P*_rhaBAD_*-RBS-*mNG*-T_BBa_B1002_ amplified with the primer pairs PF1/PF6 and PF7/PF4 from template pBBR1MCS-5-*rhaSR*-P*_rhaSR_*-P*_rhaBAD_*-*mNG*. Due to the design of the primers, *rhaS*, being the first gene of the *rhaSR* operon, was deleted in such a way that the first and last three codons of *rhaS* remained *in frame* in the plasmid, thereby in principle maintaining the original operon structure.

The plasmid pBBR1MCS-5-P*_rhaSR_*-P*_rhaBAD_*-*mNG* lacking the whole *rhaSR* operon was constructed with a DNA fragment comprising P*_rhaSR_*-P*_rhaBAD_*-RBS-*mNG*-T_BBa_B1002_ generated with the primer pair PF8/PF4 from pBBR1MCS-5-*rhaSR*-P*_rhaSR_*-P*_rhaBAD_*-*mNG* by inserting it into pBBR1MCS-5-T*_gdhM_*-MCS-T_GOX0028_.

The plasmid pBBR1MCS-5-*rhaS*-P_GOX0264_-P*_rhaBAD_*-*mNG* was constructed using three DNA fragments: The first fragment contained *rhaS* and was amplified with the primer pair PF5/PF9 from pBBR1MCS-5-*rhaS*-P*_rhaSR_*-P*_rhaBAD_*-*mNG*. The second fragment contained RBS-P_GOX0264_ and was amplified with primer pair PF10/PF11 from the genome of *G. oxydans* 621H. The third fragment contained P*_rhaBAD_*-RBS-*mNG*-T_BBa_B1002_ and was amplified with primer pair PF12/PF4 from pBBR1MCS-5-*rhaS*-P*_rhaSR_*-P*_rhaBAD_*-*mNG*.

Similarly, plasmid pBBR1MCS-5-*rhaS*-P_GOX0452_-P*_rhaBAD_*-*mNG* was constructed from three DNA fragments: Again, the first fragment contained *rhaS* and was amplified with the primer pair PF5/PF9 from pBBR1MCS-5-*rhaS*-P*_rhaSR_*-P*_rhaBAD_*-*mNG*. The second fragment contained RBS-P_GOX0452_ and was amplified with the primer pair PF13/PF14 from the genome of *G. oxydans* 621H. The third fragment contained P*_rhaBAD_*-RBS-*mNG*-T_BBa_B1002_ and was amplified with the primer pair PF15/PF4 from pBBR1MCS-5-*rhaS*-P*_rhaSR_*-P*_rhaBAD_*-*mNG*.

The plasmid pBBR1MCS-5-*mNG*-P*_rhaSR_*-P_GOX0264_-*rhaS* was created with the DNA fragment T_BBa_B1002_-*mNG*-RBS-P*_rhaBAD_* amplified with the primer pair PF16/PF17 from pBBR1MCS-5-*rhaS*-P*_rhaSR_*-P*_rhaBAD_*-*mNG* and with fragment P_GOX0264_-RBS-*rhaS* amplified with the primer pair PF18/PF19 from pBBR1MCS-5-*rhaS*-P_GOX0264_-P*_rhaBAD_*-*mNG.*

Similarly, plasmid pBBR1MCS-5-*mNG*-P*_rhaSR_*-P_GOX0452_-*rhaS* was generated from DNA fragment T_BBa_B1002_-*mNG*-RBS-P*_rhaBAD_* amplified with the primer pair PF16/PF20 from template pBBR1MCS-5-*rhaS*-P*_rhaSR_*-P*_rhaBAD_*-*mNG* and from DNA fragment P_GOX0452_-RBS-*rhaS* amplified with the primer pair PF21/PF19 from template pBBR1MCS-5-*rhaS*-P_GOX0452_-P*_rhaBAD_*-*mNG*.

The plasmid pBBR1MCS-5-*mNG*-P*_rhaSR_* was constructed with DNA fragment T_BBa_B1002_-*mNG* amplified with the primer pair PF16/PF22 and DNA fragment RBS-P*_rhaSR_*-P*_rhaBAD_* amplified with the primer pair PF23/PF24, both fragments generated from plasmid pBBR1MCS-5-*rhaS*-P*_rhaSR_*-P*_rhaBAD_*-*mNG* as PCR template. In the resulting construct pBBR1MCS-5-*mNG*-P*_rhaSR_*, the P*_rhaBAD_* region next to P*_rhaSR_* was included to retain the native P*_rhaSR_* upstream region.

For construction of plasmid pBBR1MCS-5-*rhaS*-P*_rhaSR_*-P_*rhaBAD*(+RhaS-BS)_-*mNG* containing an additional RhaS binding site (+RhaS-BS) directly downstream from the −10 region of P*_rhaBAD_*, a DNA fragment consisting of _(+RhaS-BS)_-RBS-*mNG*-T_BBa_B1002_ was amplified with the primer pair PF25/PF4 from pBBR1MCS-5-*rhaS*-P*_rhaSR_*-P*_rhaBAD_*-*mNG* and integrated into the *Eco*RI-restricted plasmid pBBR1MCS-5-*rhaS*-P*_rhaSR_*-P*_rhaBAD_*-*mNG*. The additional RhaS binding site was introduced by primer PF25.

The plasmid pBBR1MCS-5-P*_rhaSR_*-*rhaS* was constructed from plasmid pBBR1MCS-5-*rhaS*-P*_rhaSR_*-P*_rhaBAD_*-*mNG* by *Eco*RI digestion and religation. Two *Eco*RI sites were located perfectly to remove *mNG* without the need of further cloning steps.

The plasmid pBBR1MCS-5-P*_rhaT_*-*mNG* lacking *rhaS* was constructed with the DNA fragments P*_rhaT_*-RBS generated with the primer pair PF26/PF27 from the genome of *E. coli* LJ110 and the fragment *mNG*-T_BBa_B1002_ generated with the primer pair PF28/PF4 from plasmid pBBR1MCS-5-*rhaS*-P*_rhaSR_*-P*_rhaBAD_*-*mNG*.

The plasmid pBBR1MCS-5-*rhaS*-P*_rhaSR_*-P*_rhaT_*-*mNG* was created with two DNA fragments. The first DNA fragment contained *rhaS*-P*_rhaSR_* amplified with the primer pair PF5/PF29 from pBBR1MCS-5-*rhaS*-P*_rhaSR_*-P*_rhaBAD_*-*mNG*. The second DNA fragment contained P*_rhaT_*-RBS-*mNG*-T_BBa_B1002_ amplified with the primer pair PF30/PF4 from pBBR1MCS-5-P*_rhaT_*-*mNG*.

The plasmid pBBR1MCS-5-*rhaS*-P_GOX0264_-P*_rhaT_*-mNG was constructed using two fragments. The fragment containing *rhaS*-P_GOX0264_ was amplified from template pBBR1MCS-5-*rhaS*-P_GOX0264_-P*_rhaBAD_*-*mNG* with the primer pair PF5/PF34. The fragment containing P*_rhaT_*-*mNG* was amplified from template pBBR1MCS-5-*rhaS*-P*_rhaSR_*-P*_rhaT_*-*mNG* with the primer pair PF4/PF37.

The plasmid pBBR1MCS-5-*rhaS*-P_GOX0452_-P*_rhaT_*-mNG was constructed using two fragments. The fragment containing *rhaS*-P_GOX0452_ was amplified from template pBBR1MCS-5-*rhaS*-P_GOX0452_-P*_rhaBAD_*-*mNG* with the primer pair PF5/PF38. The fragment containing P*_rhaT_*-*mNG* was amplified from template pBBR1MCS-5-*rhaS*-P*_rhaSR_*-P*_rhaT_*-*mNG* with the primer pair PF4/PF39.

The plasmid pBBR1MCS-5-*rhaS*-P_GOX0264_-P_*rhaT*(−10-RhaS-BS)_-*mNG* was constructed using two fragments. The fragment containing *rhaS*-P_GOX0264_ and the 5′ part of P*_rhaT_* was amplified from template pBBR1MCS-5-*rhaS*-P_GOX0264_-P*_rhaT_*-*mNG* with the primer pair MM14/PF5. The fragment containing the remaining 3′ part of P*_rhaT_* followed by *mNG* was amplified from template pBBR1MCS-5-*rhaS*-P_GOX0264_-P*_rhaT_*-*mNG* with the primer pair MM13/PF4. The additional RhaS binding site directly downstream from the −10 region of P*_rhaT_* was created and introduced by the primers MM13 and MM14.

The plasmid pBBR1MCS-5-*rhaS*-P_GOX0452_-P_*rhaT*(−10-RhaS-BS)_-*mNG* was constructed using two fragments. The fragment containing *rhaS*-P_GOX0452_ and the 5′ part of P*_rhaT_* was amplified from template pBBR1MCS-5-*rhaS*-P_GOX0452_-P*_rhaT_*-*mNG* with the primer pair MM14/PF5. The fragment containing the remaining 3′ part of P*_rhaT_* followed by *mNG* was amplified from template pBBR1MCS-5-*rhaS*-P_GOX0452_-P*_rhaT_*-*mNG* with the primer pair MM13/PF4. The additional RhaS binding site directly downstream from the −10 region of P*_rhaT_* was created and introduced by the primers MM13 and MM14.

The plasmid pBBR1MCS-5-*rhaS*-P_GOX0264_-P_*rhaT*(TSS-RhaS-BS)_-*mNG* was constructed using two fragments. The fragment containing *rhaS*-P_GOX0264_ and the 5′ part of P*_rhaT_* was amplified from template pBBR1MCS-5-*rhaS*-P_GOX0264_-P*_rhaT_*-*mNG* with the primer pair MM16/PF5. The fragment containing the remaining 3′ part of P*_rhaT_* followed by *mNG* was amplified from template pBBR1MCS-5-*rhaS*-P_GOX0264_-P*_rhaT_*-*mNG* with the primer pair MM15/PF4. The additional RhaS binding site directly downstream from the *E. coli* transcriptional start of P*_rhaT_* was created and introduced by the primers MM15 and MM16.

The plasmid pBBR1MCS-5-*rhaS*-P_GOX0452_-P_*rhaT*(TSS-RhaS-BS)_-*mNG* was constructed using two fragments. The fragment containing *rhaS*-P_GOX0452_ and the 5′ part of P*_rhaT_* was amplified from template pBBR1MCS-5-*rhaS*-P_GOX0452_-P*_rhaT_*-*mNG* with the primer pair MM16/PF5. The fragment containing the remaining 3′ part of P*_rhaT_* followed by *mNG* was amplified from template pBBR1MCS-5-*rhaS*-P_GOX0452_-P*_rhaT_*-*mNG* with the primer pair MM15/PF4. The additional RhaS binding site directly downstream from the *E. coli* transcriptional start of P*_rhaT_* was created and introduced by the primers MM15 and MM16.

The plasmid pKOS6b-igr3::*mNG* for genomic labelling of *G. oxydans* 621H by *mNG* under control of P_*rhaBAD*(+RhaS-BS)_ in igr3 was constructed with three fragments. The upstream and downstream flanking regions of igr3 were amplified from genomic DNA of *G. oxydans* 621H with the primer pairs PF31/PF32 and PF33/PF34, respectively. The fragment containing P_*rhaBAD*(+RhaS-BS)_-*mNG* was amplified from plasmid pBBR1MCS-5-*rhaS*-P*_rhaSR_*-P_*rhaBAD*(+RhaS-BS)_-*mNG* with the primer pair PF35/PF36.

The plasmid pKOS6b-igr2::P_GOX0264_-*rhaS* for genomic integration of a P_GOX0264_-*rhaS* copy into igr2 of *G. oxydans mNG* was constructed with three fragments. The upstream and downstream flanking regions of igr2 were amplified from genomic DNA of *G. oxydans* 621H with the primer pairs PF40/PF41 and PF42/PF43, respectively. The fragment containing P_GOX0264_-*rhaS* was amplified from plasmid pBBR1MCS-5-*rhaS*-P_GOX0264_-P*_rhaBAD_*-*mNG* with the primer pair PF44/PF45.

The plasmid pKOS6b-igr2::P*_rhaSR_*-*rhaS* for genomic integration of a P*_rhaSR_*-*rhaS* copy into igr2 of *G. oxydans mNG* was constructed with three fragments. The upstream and downstream flanking regions of igr2 were amplified from genomic DNA of *G. oxydans* 621H with the primer pairs PF40/PF46 and PF42/PF43, respectively. The fragment containing P*_rhaSR_*-*rhaS* was amplified from plasmid pBBR1MCS-5-*rhaS*-P*_rhaSR_*-P*_rhaBAD_*-*mNG* with the primer pair PF44/PF47.

The plasmid pKOS6b-igr1::P_GOX0264_-*rhaS* for genomic integration of a P_GOX0264_-*rhaS* copy into igr1 of *G. oxydans mNG* igr2::P_GOX0264_-*rhaS* was constructed with three fragments. The upstream and downstream flanking regions of igr1 were amplified from genomic DNA of *G. oxydans* 621H with the primer pairs MH3/MH10 and MH6/MH9, respectively. The fragment containing P_GOX0264_-*rhaS* was amplified from plasmid pBBR1MCS-5-*rhaS*-P_GOX0264_-P*_rhaBAD_*-*mNG* with the primer pair MH4/MH5.

The plasmid pKOS6b-igr1::P*_rhaSR_*-*rhaS* for genomic integration of a P*_rhaSR_*-*rhaS* copy into igr1 of *G. oxydans mNG* igr2::P_GOX0264_-*rhaS* was constructed with three fragments. The upstream and downstream flanking regions of igr1 were amplified from genomic DNA of *G. oxydans* 621H with the primer pairs MH7/MH10 and MH6/MH9, respectively. The fragment containing P*_rhaSR_*-*rhaS* was amplified from plasmid pBBR1MCS-5-*rhaS*-P*_rhaSR_*-P*_rhaBAD_*-*mNG* with the primer pair MH5/MH8.

### Construction and selection of genomically modified *Gluconobacter oxydans* strains

Integrations of expression cassettes into the genome of *G. oxydans* 621H and selection of excised plasmid backbones were carried out using pKOS6b plasmid derivatives and counterselection by cytosine deaminase, encoded by *codA* from *E. coli*, in the presence of the fluorinated pyrimidine analogue 5-fluorocytosine (FC). The cytosine deaminase converts nontoxic FC to toxic 5-fluorouracil, which is channeled into the metabolism by the uracil phosphoribosyltransferase, encoded by the chromosomal *upp* gene of *Gluconobacter*. The details of the method are described elsewhere (Kostner et al., 2013). According to this method, strain *G. oxydans mNG* was constructed and selected from *G. oxydans* 621H using the plasmid pKOS6b-igr3::*mNG*. The *G. oxydans* strains *mNG* igr2::P_GOX0264_-*rhaS* and *mNG* igr2::P*_rhaSR_*-*rhaS* were constructed and selected from *G. oxydans mNG* using the plasmids pKOS6b-igr2::P_GOX0264_-*rhaS* and pKOS6b-igr2::P*_rhaSR_*-*rhaS*, respectively. The *G. oxydans* strains *mNG* igr1::P_GOX0264_-*rhaS* igr2::*rhaS* and *mNG* igr1::P*_rhaSR_*-*rhaS* igr2::*rhaS* were constructed and selected from *G. oxydans mNG* igr2::P_GOX0264_-*rhaS* using the plasmids pKOS6b-igr1::P_GOX0264_-*rhaS* and pKOS6b-igr1::P*_rhaSR_*-*rhaS*, respectively.

### Measurements of fluorescence protein

The regulation and relative strength of the promoters on constructed plasmids was monitored in *G. oxydans* by means of expressing *mNG* encoding the fluorescent reporter protein mNG (Shaner et al., 2013). For analysis of *mNG* expression with various promoters by mNG signals, *G. oxydans* cultures were supplemented with l-rhamnose at the indicated concentrations (w/v) using a 40% (w/v) stock solution. Equal volumes of medium were added to non-supplemented reference cultures. Throughout the cultivation, growth (OD_600_) and fluorescence emission were monitored in intervals using a spectrophotometer (UV-1800, Shimadzu) and an Infinite M1000 PRO Tecan reader (*λ*_ex_ 504 nm/*λ*_em_ 517 nm, ex/em bandwidth 5 nm, infinite M1000 PRO Tecan). For microscale BioLector cultivations, overnight starter cultures were used to inoculate 800 μl batches of d-mannitol medium in 48-well Flowerplates^®^ (m2p-labs) to an initial OD_600_ of 0.3. Sealed with disposable foil (m2p-labs), plates were cultivated for 24 h at 1,200 rpm, 85% humidity and 30°C. Growth was monitored in each well as backscattered light at 620 nm (A_620 nm_) and protein fluorescence was monitored as emission (*λ*_ex_ 510 nm/*λ*_em_ 532 nm). For backscatter signal amplification, gain 20 was applied. Signal amplification of fluorescence emission varied (gain 40–70) and is indicated in the figure legends. All BioLector data shown in a diagram were measured in the same run of a growth experiment.

### Cell flow cytometer analysis

For single cell analysis, a FACSAria™ cell sorter controlled by FACSDiva 8.0.3 software (BD Biosciences) was used to analyze the mNG reporter protein signals in *G. oxydans* 621H harboring either plasmid pBBR1MCS-5-*rhaS*-P*_rhaSR_*-P*_rhaBAD_*-*mNG* or pBBR1MCS-5-*rhaS*-P*_rhaSR_*-P*_rhaT_*-*mNG*. The FACS was operated with a 70 μm nozzle and run with a sheath pressure of 70 psi. The forward scatter (FSC) and side scatter (SSC) were recorded as small-angle scatter and orthogonal scatter, respectively, by means of a 488 nm solid blue laser beam. For analysis, only particles/events above 200 a.u. for FSC-H and above 300 a.u. for SSC-H as the thresholds were considered. The mNG fluorescence emission was detected from the SSC through the combination of a 502 nm long-pass and 530/30 nm band-pass filter. Prior to data acquisition, the FSC-A vs. SSC-A plot was employed to gate the population and to exclude signals originating from cell debris or electronic noise. In a second and third gating step, from the resulting population, the SSC-H signal was plotted against the SSC-W signal and this population was subsequently gated in a FSC-H vs. FSC-W plot to exclude doublets. From this resulting singlet population, 100,000 events were recorded at a rate of <10,000 events/s for fluorescence data acquisition. For data analysis and visualization of all gated events (*n* = 100,000) FlowJo 10.7.2 for Windows (FlowJo, LLC) was applied.

### l-Rhamnose biotransformation test assay and GC-TOF-MS analysis

*G. oxydans* cells were grown to an OD_600_ of 1.3, centrifuged (4,000 × *g*, 5 min) and washed twice with 50 mM phosphate buffer (pH 6). After the second washing step, cells were resuspended in biotransformation buffer (6.6 g L^−1^ Na_2_HPO_4_, 3 g L^−1^ KH_2_PO_4_, 1 g L^−1^ NH_4_Cl, 0.5 g L^−1^ NaCl, 0.49 g L^−1^ MgSO_4_, 0.02 g L^−1^ CaCl_2_) supplemented with 2% (w/v) l-rhamnose and incubated for 24 h at 30°C and 200 rpm. Then, the cells were removed from the buffer (4,000 × *g*, 5 min) and the supernatant was used for analysis by gas chromatography (Agilent 6,890 N, Agilent Technologies) coupled to a Waters Micromass GCT Premier high-resolution time-of-flight mass spectrometer (Waters). Sample handling for derivatization, GC-TOF-MS operation, and peak identification were carried out as described (Paczia et al., 2012). As a control, samples from biotransformation buffer with l-rhamnose and without cells as well as biotransformation buffer without l-rhamnose yet with cells were prepared.

### Total DNA extraction, library preparation, illumina sequencing, and data analysis

Total DNA was purified from a culture aliquot using a NucleoSpin Microbial DNA Mini kit (MACHEREY–NAGEL). DNA concentrations were measured using a Qubit 2.0 fluorometer (Thermo Fisher Scientific). Illumina sequencing and data analysis of the indicated P*_rhaBAD_* DNA sample was carried out as described (Fricke et al., 2021b). For the read mapping, the improved genome sequence from *G. oxydans* 621H and the indicated P*_rhaBAD_* plasmid sequence were used (Kranz et al., 2017).

### Determination of transcriptional starts

*G. oxydans* cells carrying plasmid pBBR1MCS-5-*rhaS*-P*_rhaSR_*-P_*rhaBAD*(+RhaS-BS)_-*mNG* were cultivated in shake flasks with 50 ml complex d-mannitol medium. Cells were harvested at OD_600_ of 1.5 in the mid-exponential phase and total RNA was extracted as described (Kranz et al., 2018). The RNA sample was sent to the company Vertis Biotechnology AG (Germany) for further sample processing and data generation. For Cappable-seq RNA, the RNA sample was enriched by capping of the 5′ triphosphorylated RNA with 3′-desthiobiotin-TEG-guanosine 5′ triphosphate (DTBGTP; NEB) using the vaccinia capping enzyme (VCE; NEB) for reversible binding of biotinylated RNA species to streptavidin. Then, streptavidin beads were used to capture biotinylated RNA species followed by elution to obtain highly enriched 5′ fragment of the primary transcripts. The Cappable enriched RNA sample was poly(A)-tailed using poly(A) polymerase. In order to remove residual 5’-P-ends, the RNA was treated with Antarctic Phosphatase (NEB). Then, the 5’-PPP cap structures were converted to 5’-P using the RppH enzyme (NEB). Afterwards, an RNA adapter was ligated to the newly formed 5′-monophosphate structures. First-strand cDNA synthesis was performed using an oligo(dT)-adapter primer and the MMLV reverse transcriptase. The resulting cDNA was PCR-amplified to about 10–20 ng/μl using a high fidelity DNA polymerase. For Illumina sequencing, 100–300 bp long 5′ fragments were isolated from the full-length cDNA. For this purpose the cDNA preparation was fragmented and the 5’-cDNA fragments were then bound to streptavidin magnetic beads. The bound cDNAs were blunted and the 3’ Illumina sequencing adapter was ligated to the 3′ ends of the cDNA fragments. The bead-bound cDNAs were finally PCR-amplified. The library was sequenced on an Illumina NextSeq 500 system using 75 bp read length. The fastq file output was used for data analysis with CLC Genomics Workbench (v21.0.3). Imported reads were trimmed and quality filtered. Passed reads were used for strand-specific mapping to the *G. oxydans* genome and the pBBR1MCS-5-*rhaS*-P*_rhaSR_*-P_*rhaBAD*(+RhaS-BS)_-*mNG* plasmid sequence using the RNA-seq analysis tool implemented in the CLC software. Read mapping settings used were 80% length fraction and 80% similarity fraction. The starts of mapped reads and total nucleotide coverage according to the mappings were used to assess transcriptional starts on the expression plasmid with the promoters P*_rhaSR_* and P_*rhaBAD*(+RhaS-BS)_.

## Results

### l-Rhamnose does not affect growth and is not oxidized by *Gluconobacter oxydans* 621H

The RhaSR-P*_rhaBAD_* system from *E. coli* responds to the monosaccharide rhamnose in the uncommon l conformation, which is similar to the AraC-P*_araBAD_* system and its effector l-arabinose. Like l-arabinose, the inducer l-rhamnose needs to enter the cell to interact with its targeted regulators RhaR and RhaS (Tobin and Schleif, 1987). In contrast to l-arabinose, which is readily oxidized by *Gluconobacter* already in the periplasm (Peters et al., 2013; Fricke et al., 2022), for more than 90% of the strains of the genus *Gluconobacter* no acid formation from l-rhamnose has been reported (Kersters et al., 1990). *G. oxydans* 621H whole-cell enzyme activity assays using the artificial electron acceptor DCPIP also revealed no detectable activity with l-rhamnose as substrate (Peters et al., 2013). To exclude a hitherto unrecognized consumption or oxidation of the inducer l-rhamnose by *G. oxydans* 621H, we carried out biotransformation assays followed by GC-TOF-MS analysis, and a growth experiment.

The results confirmed that *G. oxydans* does not consume or oxidize l-rhamnose. In the GC-TOF-MS analysis, no new peaks were detected in 24 h samples, and the areas of the GC-TOF peaks assigned to l-rhamnose were very similar for the samples at 0 h and after 24 h (Supplementary Table S2; Supplementary Figure S2). Hence, if at all, l-rhamnose is degraded or converted by strain 621H so slowly that this effector is hardly diminished during potential applications. To check if l-rhamnose somehow affects the growth of *G. oxydans* 621H, we added l-rhamnose to the complex medium. With 1% (w/v) l-rhamnose instead of d-mannitol, there was no growth of *G. oxydans* 621H and the initial start OD_600_ of 0.04 did not change within 24 h (Supplementary Figure S3). In 4% (w/v) d-mannitol medium supplemented with 1% (w/v) l-rhamnose, the strain 621H grew very similar and without a significant difference compared to the growth in the d-mannitol complex medium without l-rhamnose supplement. Furthermore, with and without l-rhamnose the initial pH 6 of the growth medium was acidified to pH 4.3 after 24 h, suggesting no relevant oxidation of l-rhamnose to a corresponding acid. Therefore, there was no negative or supportive effect of l-rhamnose on the growth of *G. oxydans* 621H up to 1% (w/v).

### In *Gluconobacter oxydans*, P*_rhaBAD_* from *Escherichia coli* is repressed in the presence of l-rhamnose

**Figure 1:**
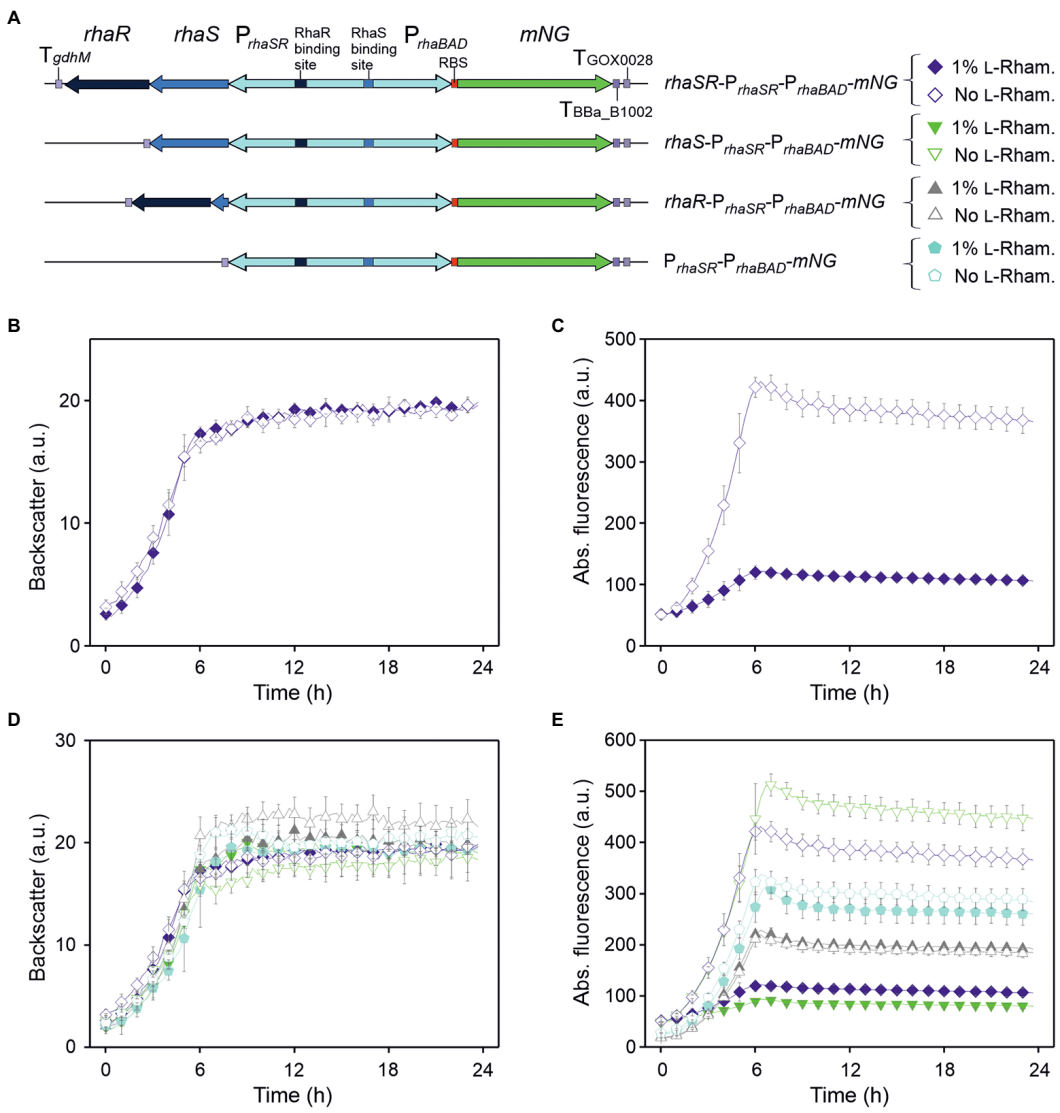
pBBR1MCS-5-based expression plasmids and analysis of the regulation of the RhaSR-P*_rhaBAD_* system from *Escherichia coli* in *G. oxydans* 621H. **(A)** Schematic illustration of the plasmid variants with reporter gene *mNG* used to test l-rhamnose-dependent regulation of P*_rhaBAD_*-derived expression in the presence and absence of RhaS and RhaR. T*_gdhM_*: terminator sequence of *gdhM* (GOX0265); T_GOX0028_: terminator sequence of GOX0028. The RBS 5′-AGGAGA was inserted in the 3′ region of P*_rhaBAD_* upstream from *mNG*. **(B)** Growth according to backscatter and **(C)** absolute mNG fluorescence in *G. oxydans* carrying plasmid pBBR1MCS-5-*rhaSR*-P*_rhaSR_*-P*_rhaBAD_*-*mNG* grown in d-mannitol medium without and with 1% (w/v) l-rhamnose in BioLector microscale. **(D)** Growth according to backscatter and **(E)** absolute mNG fluorescence of *G. oxydans* carrying either plasmid pBBR1MCS-5-*rhaSR*-P*_rhaSR_*-P*_rhaBAD_*-*mNG*, or a plasmid lacking either *rhaR*, or *rhaS*, or both *rhaSR*. Cells were grown in microscale (BioLector) in d-mannitol medium without and with 1% (w/v) l-rhamnose. All data represent mean values and standard deviation from two biological replicates (clones) with three technical replicates each. BioLector settings: backscatter gain 20, fluorescence gain 50.

First, we tested the inducibility of P*_rhaBAD_* in *G. oxydans* by constructing a pBBR1MCS-5-based plasmid placing all the genetic elements in the same order as in *E. coli*. The *rhaSR* operon was under the control of its native promoter P*_rhaSR_* in divergent orientation to P*_rhaBAD_*. The fluorescent reporter mNeonGreen (mNG) was used to measure the P*_rhaBAD_*-controlled expression by placing the *mNG* gene downstream from P*_rhaBAD_*. On the plasmid, the elements *rhaSR*-P*_rhaSR_*-P*_rhaBAD_*-*mNG* were flanked by three terminators, T*_gdhM_* downstream from *rhaR*, and T_BBa_B1002_ and T_GOX0028_ downstream from *mNG* (Figure 1A). Furthermore, downstream from the native ribosome binding site (RBS) present in P*_rhaBAD_* the RBS 5′-AGGAGA was inserted upstream from *mNG*. This RBS appeared strong in *G. oxydans* and was also used in the regulatable AraC-P*_araBAD_* and TetR-P*_tet_* expression systems (Fricke et al., 2020, 2021b). The inducibility of the resulting plasmid pBBR1MCS-5-*rhaSR*-P*_rhaSR_*-P*_rhaBAD_*-*mNG* was tested in *G. oxydans* 621H with 1% (w/v) l-rhamnose. Overnight pre-cultures were split to inoculate main cultures in d-mannitol medium with and without l-rhamnose. Growth and mNG fluorescence was monitored in a BioLector.

As expected from the previous growth tests in shake flasks, all BioLector microscale cultures exhibited very similar growth regardless of l-rhamnose supplementation (Figure 1B). However, surprisingly and contrary to our expectation, the mNG fluorescence of the cultures without l-rhamnose strongly increased during growth and peaked ~6 h after inoculation when cells entered the stationary phase, while in cultures with l-rhamnose a much lower level of mNG fluorescence (~28%) was observed (Figure 1C). Thus, *mNG* expression from P*_rhaBAD_* appeared to be strongly repressed in the presence of l-rhamnose, suggesting that in *G. oxydans* the responsiveness of the RhaSR-P*_rhaBAD_* system is inverted compared to *E. coli*. Furthermore, according to the absolute mNG fluorescence in the absence of l-rhamnose, the promoter P*_rhaBAD_* appeared to be very strong in *G. oxydans* compared to P*_araBAD_* and P*_tet_* (Fricke et al., 2020, 2021b).

To test whether the *rhaSR*-P*_rhaBAD_*-*mNG* expression plasmid shows l-rhamnose-inducibility in *E. coli*, the plasmid-carrying *E. coli* S17-1 used for transformation of *G. oxydans* was tested. As expected, in LB medium supplemented with 1% (w/v) l-rhamnose, the mNG fluorescence was ~2,200-fold higher compared to the mNG fluorescence in cultures without l-rhamnose (data not shown). To verify that the reversed responsiveness of RhaSR-P*_rhaBAD_* indeed was observed in *G. oxydans* 621H carrying the intended plasmid without mutations possibly acquired later during growth, *G. oxydans* cells of an induced culture were harvested at the end of the cultivation (24 h) for isolation of total DNA and Illumina sequencing. The read data analysis excluded unexpected contamination of the culture, since 99.48% of 1,402,738 trimmed and quality-filtered reads mapped to the updated reference sequences of the *G. oxydans* 621H genome (88-fold coverage), the 5 endogenous plasmids, and the *mNG* expression plasmid with *rhaSR*-P*_rhaSR_*-P*_rhaBAD_*-*mNG* (1,011-fold coverage; Kranz et al., 2017). Besides, the sequencing results corroborated three DNA point mutations in *rhaSR* already observed before by Sanger sequencing when checking the insert of the plasmid after cloning in *E. coli*. In *rhaR* there was the silent mutation of CGC to CGT (Arg56). In *rhaS* there was the silent mutation of CTG to CTT (Leu166) and the mutation of GGG to TGG resulting in the exchange Gly136Trp in RhaS. All three mutations were present already on the plasmid when it was cloned in *E. coli*. To exclude an effect of these mutations on the reversed responsiveness only in *G. oxydans*, the plasmid was cloned again using a new *rhaS* DNA template from *E. coli* MG1655. This plasmid lacked the two point mutations in *rhaS* and also showed the reversed responsiveness in *G. oxydans* 621H with the same extent of repression (data not shown). Thus, the DNA point mutations in *rhaS* did not affect the regulatory properties of the system in *G. oxydans*. In summary, these results showed that in contrast to *E. coli* the P*_rhaBAD_* promoter is repressed in *G. oxydans* in the presence of l-rhamnose.

### RhaS is responsible for l-rhamnose-dependent repression of P*_rhaBAD_* in *Gluconobacter oxydans*

To analyze whether RhaS and/or RhaR, or an interfering endogenous *G. oxydans* protein is responsible for the reversed responsiveness of the RhaSR-P*_rhaBAD_* system, we constructed derivatives of the expression plasmid either lacking in-frame a substantial part of *rhaS*, or lacking *rhaR*, or lacking both genes, yet keeping all the elements upstream and downstream from *rhaS* and *rhaR* (Figure 1A). *G. oxydans* clones carrying one of these plasmid derivatives were grown in d-mannitol medium without and with 1% (w/v) l-rhamnose and cultivated in a BioLector to monitor growth and mNG fluorescence. Regardless of the plasmid used, all *G. oxydans* cultures exhibited very similar growth with and without l-rhamnose (Figure 1D). The differences in mNG fluorescence with and without l-rhamnose clearly indicated that RhaS alone is either directly or indirectly responsible for the regulation of P*_rhaBAD_*. All clones with the plasmid lacking only *rhaS* exhibited a moderate maximal mNG fluorescence after ~6 h (220–228 a.u.), regardless of l-rhamnose supplementation (Figure 1E). The clones with the plasmid lacking both *rhaS* and *rhaR* also showed no response of the mNG fluorescence to l-rhamnose, yet the maximal mNG fluorescence was 50% higher compared to the plasmid still containing *rhaR*. Without *rhaSR*, the mNG signals of all clones peaked at 6 h and reached a higher intensity (314–338 a.u.), suggesting a general negative effect of RhaR on the P*_rhaBAD_* activity regardless of the presence or absence of l-rhamnose. This is in line with the observation that with the plasmid lacking only *rhaR*, expression from P*_rhaBAD_* increased in the absence of l-rhamnose by ~20% (513 a.u.) compared to the plasmid with both regulator genes (431 a.u.). Furthermore, with 1% (w/v) l-rhamnose the *mNG* expression from P*_rhaBAD_* was more reduced with the *rhaS*-P*_rhaBAD_* construct (94 a.u.) than with the *rhaSR*-P*_rhaBAD_* construct (122 a.u.).

In summary, these data indicated that RhaS activates the P*_rhaBAD_* promoter in the absence of l-rhamnose and represses P*_rhaBAD_* in the presence of l-rhamnose, and thus is exerting a dual role in *G. oxydans* (Figure 1E).

### In the absence of l-rhamnose P*_rhaBAD_* activity is stimulated by RhaS

**Figure 2:**
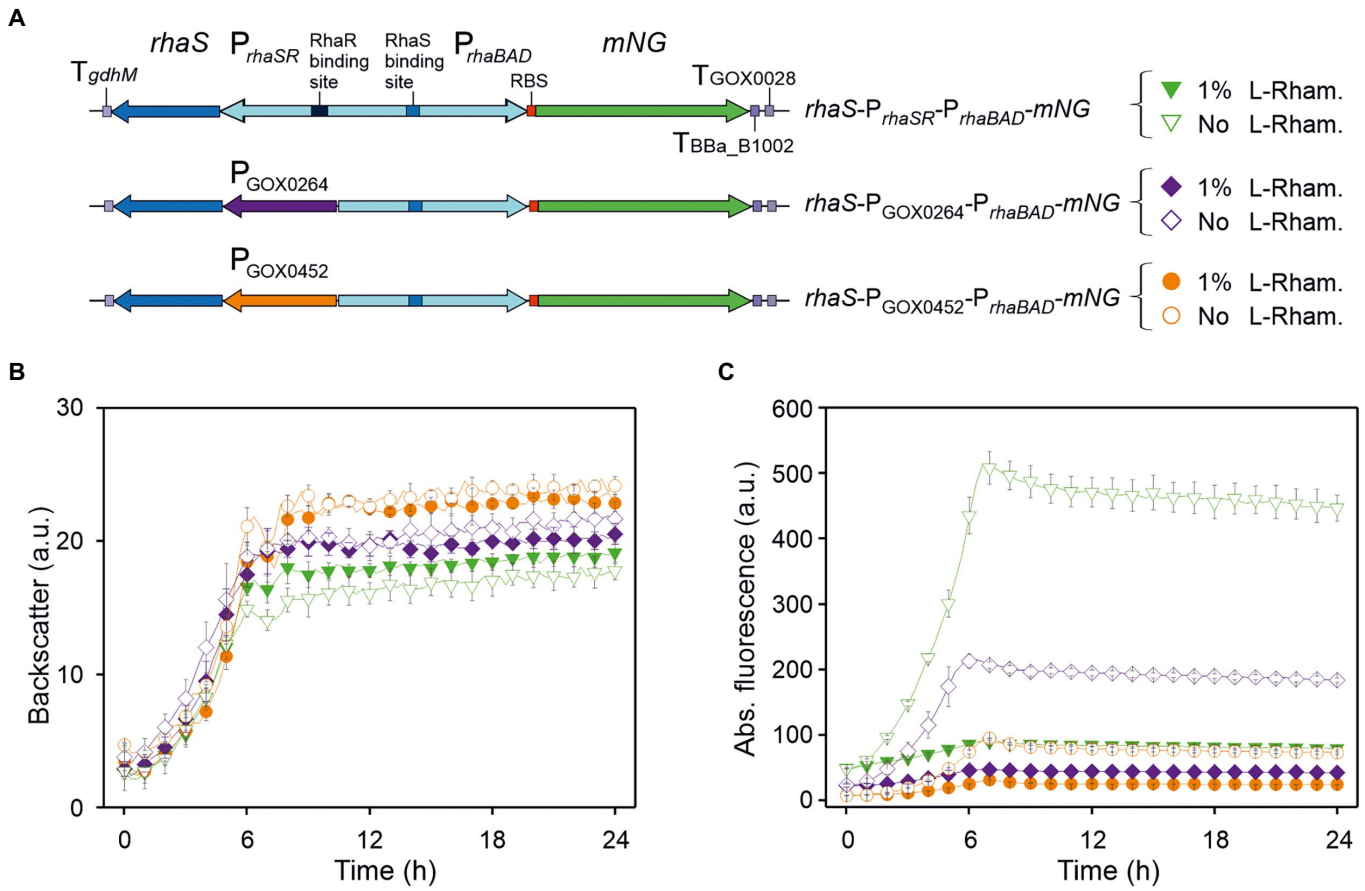
Performance of P*_rhaBAD_*-derived *mNG* expression in dependence of *rhaS* expression and presence of l-rhamnose. **(A)** Schematic illustration of the plasmid variants for constitutive expression of *rhaS* from the strong and moderate *G. oxydans* promoters P_GOX0264_ and P_GOX0452_. T*_gdhM_*: terminator sequence of *gdhM* (GOX0265); T_GOX0028_: terminator sequence of GOX0028. RBS: 5′-AGGAGA inserted in the 3′ region of P*_rhaBAD_* upstream from *mNG*. **(B)** Growth in d-mannitol medium according to backscatter and **(C)** absolute mNG fluorescence of *G. oxydans* 621H carrying either plasmid pBBR1MCS-5-*rhaS*-P*_rhaSR_*-P*_rhaBAD_*-*mNG*, or pBBR1MCS-5-*rhaS*-P_GOX0264_-P*_rhaBAD_*-*mNG*, or pBBR1MCS-5-*rhaS*-P_GOX0452_-P*_rhaBAD_*-*mNG*. Repression of *mNG* expression from P*_rhaBAD_* was tested with 1% (w/v) l-rhamnose. Data represent mean values and standard deviation from two biological replicates (clones) with three technical replicates each. BioLector settings: backscatter gain 20, fluorescence gain 50.

The clear differences in the mNG fluorescence observed with the previous plasmid derivatives with or without *rhaS* suggested that RhaS activates P*_rhaBAD_* in the absence of l-rhamnose in *G. oxydans*. If so, the apparent strength of P*_rhaBAD_* in the absence of l-rhamnose could partially be tuned by the strength of *rhaS* expression. To test this and the resulting down-regulation of P*_rhaBAD_*-derived *mNG* expression in the presence of l-rhamnose starting then from different initial expression levels, we constructed derivatives of pBBR1MCS-5-*rhaS*-P*_rhaSR_*-P*_rhaBAD_*-*mNG* expressing *rhaS* constitutively either from the *G. oxydans* promoter P_GOX0264_ or P_GOX0452_ (Figure 2A). P_GOX0264_ and P_GOX0452_ have been shown to be strong and moderate promoters in *G. oxydans*, respectively (Kallnik et al., 2010). With the resulting plasmids pBBR1MCS-5-*rhaS*-P_GOX0264_-P*_rhaBAD_*-*mNG* and pBBR1MCS-5-*rhaS*-P_GOX0452_-P*_rhaBAD_*-*mNG*, the *mNG* expression was compared to that with pBBR1MCS-5-*rhaS*-P*_rhaSR_*-P*_rhaBAD_*-*mNG* in microscale BioLector cultivations (Figure 2B). Without l-rhamnose, constitutive expression of *rhaS* from P_GOX0264_ reduced P*_rhaBAD_*-derived *mNG* expression by more than half and from this latter level the *mNG* expression was reduced by half when expressing *rhaS* from P_GOX0452_ (Figure 2C). Thus, expression of *rhaS* from its native promoter P*_rhaSR_* led to the highest P*_rhaBAD_*-derived mNG signals (514 a.u. after ~7 h) in the absence of l-rhamnose. These results suggested that P*_rhaSR_* is a very strong promoter *per se*, or because it is positively auto-regulated by RhaS. However, the RhaS protein was reported to severely aggregate when overexpressed (Wickstrum et al., 2010), and biochemical analysis of RhaS binding to the promoter DNA had not been possible due to the extreme insolubility of the overproduced RhaS protein (Egan and Schleif, 1994). Therefore, it appears more likely that P*_rhaSR_* is a weak promoter also in *G. oxydans* resulting in sufficient levels of functional RhaS protein activating P*_rhaBAD_*, while stronger *rhaS* expression *via* P_GOX0264_ and P_GOX0452_ likely resulted in aggregated non-functional RhaS protein.

In the presence of 1% (w/v) l-rhamnose, the strong *mNG* expression obtained with *rhaS*-P*_rhaSR_*-P*_rhaBAD_*-*mNG* was reduced by ~82% (from 514 to 90 a.u.). The *mNG* expression obtained with *rhaS*-P_GOX0264_-P*_rhaBAD_*-*mNG* and with *rhaS*-P_GOX0452_-P*_rhaBAD_*-*mNG* was reduced by 77% (from 212 to 48 a.u.) and by 68% (from 95 to 30 a.u.), respectively (Figure 2C).

### P*_rhaSR_* is weak in *Gluconobacter oxydans*, stimulated by RhaS and further stimulated by l-rhamnose

To check the strength of P*_rhaSR_* in *G. oxydans* and the influence of RhaS on P*_rhaSR_* activity, we created plasmids with *mNG* under the control of P*_rhaSR_* and with *rhaS* under the control of the constitutive promoters P_GOX0452_ or P_GOX0264_, or lacking *rhaS* (Supplementary Figure S4A). The respective *G. oxydans* strains were cultivated in a BioLector and showed similar growth (Supplementary Figure S4B). In the absence of l-rhamnose, moderate P_GOX0452_-derived *rhaS* expression resulted in a similar low *mNG* expression from P*_rhaSR_* as without *rhaS*, while the stronger P_GOX0264_-derived *rhaS* expression resulted in a two-fold higher *mNG* expression from P*_rhaSR_*, suggesting a positive effect of the RhaS level on P*_rhaSR_* activity (Supplementary Figures S4C,D). In the presence of l-rhamnose, *mNG* expression from P*_rhaSR_* was always increased with *rhaS*, while there was no effect by l-rhamnose when *rhaS* was absent. With moderate *rhaS* expression in *G. oxydans* harboring pBBR1MCS -5-*mNG-*P*_rhaSR_*-P_GOX0452_*-rhaS*, the mNG fluorescence increased ~2.5-fold from 74 to 189 a.u. with 1% (w/v) l-rhamnose. This l-rhamnose-dependent increase was less pronounced with *rhaS* under control of the stronger P_GOX0264_ where the RhaS level was expected to be higher. Here, the mNG fluorescence increased only 1.3-fold from 144 to 187 a.u. (Supplementary Figure S4C). Together, P*_rhaSR_* is also stimulated by RhaS in the absence of l-rhamnose, yet in contrast to the repressed P*_rhaBAD_* promoter, P*_rhaSR_* is further activated by RhaS in the presence of l-rhamnose.

### Repression of P*_rhaBAD_* is sensitive to low l-rhamnose levels and is homogeneous

**Figure 3:**
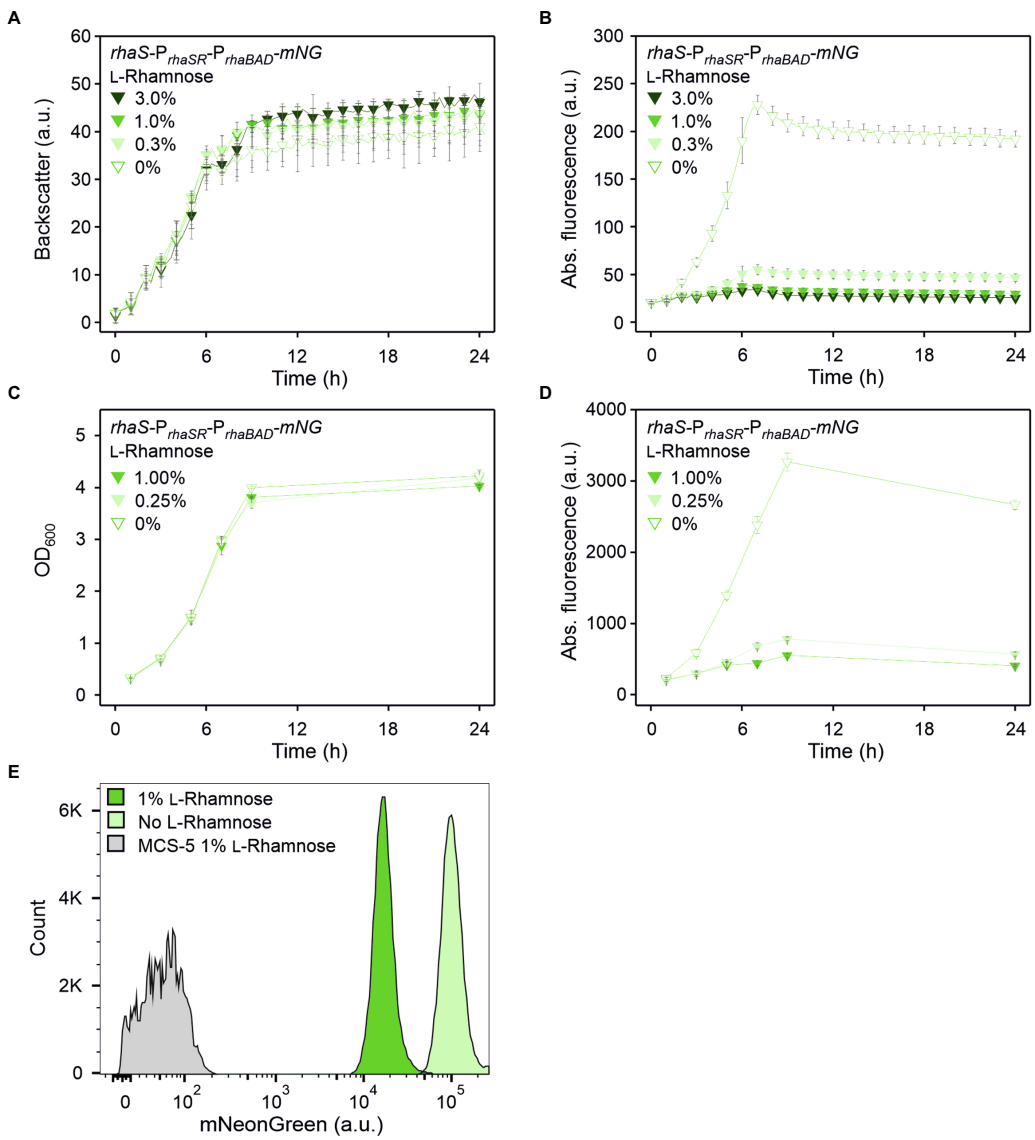
RhaS-dependent repression of P*_rhaBAD_* in *G. oxydans* in the presence of l-rhamnose. **(A)** Growth according to backscatter and **(B)** absolute mNG fluorescence in *G. oxydans* 621H with plasmid pBBR1MCS-5-*rhaS*-P*_rhaSR_*-P*_rhaBAD_-mNG* in microscale BioLector cultivations without or with l-rhamnose (w/v) as indicated. BioLector settings: backscatter gain 20, fluorescence gain 40. **(C)** Growth (OD_600_) and **(D)** absolute mNG fluorescence in *G. oxydans* 621H with plasmid pBBR1MCS-5-*rhaS*-P*_rhaSR_*-P*_rhaBAD_*-*mNG* in shake flask cultivations without and with l-rhamnose (w/v) as indicated. mNG fluorescence was measured in a Tecan reader (gain 60). Data represent mean values and standard deviation from two biological replicates (clones) with three technical replicates each. **(E)** FACS analysis of *G. oxydans* 621H with plasmid pBBR1MCS-5-*rhaS*-P*_rhaSR_*-P*_rhaBAD_*-*mNG* or empty vector pBBR1MCS-5 as a control (MCS-5). Cells were grown in shake flasks with d-mannitol medium without and with 1% (w/v) l-rhamnose. FACS analysis was performed 7 h after inoculation (induction). Total counts per sample represent 100,000 events.

Since from all tested plasmid variants the one lacking *rhaR* and containing *rhaS* under the control of its native auto-regulated P*_rhaSR_* promoter exhibited the highest P*_rhaBAD_* activity in the absence of l-rhamnose and the highest grade of repression in the presence of l-rhamnose, the construct pBBR1MCS-5-*rhaS*-P*_rhaSR_*-P*_rhaBAD_*-*mNG* was analyzed further. The sensitivity of repression and residual *mNG* expression was tested in d-mannitol medium with 0.3%, 1% and 3% (w/v) l-rhamnose in a BioLector (Figures 3A,B). Already 0.3% (w/v) l-rhamnose strongly reduced the mNG fluorescence after ~7 h by 75% (225 vs. 55 a.u.). This indicated that the RhaS-P*_rhaBAD_* system is quite sensitive and already low l-rhamnose concentrations should enable a tuning of target gene repression. Supplementation with 1% and 3% (w/v) l-rhamnose reduced mNG fluorescence by 83% (38 a.u.) and 85% (34 a.u.), respectively. This suggested that already 1% (w/v) l-rhamnose was sufficient to reach almost maximal possible repression of plasmid-based P*_rhaBAD_* copies in *G. oxydans*.

This responsiveness of P*_rhaBAD_*-based expression toward relatively low l-rhamnose concentrations was also observed in shake flask cultivations. When grown in 50 ml d-mannitol medium supplemented with 0.25% l-rhamnose, the mNG fluorescence was reduced to 24% (from 3,267 to 783 a.u.) after 9 h (Figures 3C,D). In shake flask cultures with 1% (w/v) l-rhamnose, the mNG fluorescence was reduced to 17% (from 3,267 to 553 a.u.).

Flow cytometry was used to analyze the repression of P*_rhaBAD_*-derived *mNG* expression on the single cell level. In the absence of l-rhamnose, 7 h after inoculation 95.5% of the analyzed cells showed strong mNG fluorescence (~100,000 a.u.), while when grown with 1% (w/v) l-rhamnose, 96.4% of the analyzed cells showed a 89% reduced fluorescence (~11,000 a.u.; Figure 3E). Thus, the results of this FACS analysis are in line with the results of the BioLector and Tecan reader (shake flasks) measurements. Additionally, the FACS analysis demonstrated a high population homogeneity in both conditions.

### An additional RhaS binding site directly downstream from the −10 region doubled the P*_rhaBAD_*-derived expression strength and the dynamic range of repression

In an attempt to reduce the residual expression from P*_rhaBAD_* in the presence of l-rhamnose and achieve complete repression, and to possibly lower the l-rhamnose concentrations required, we constructed and tested a plasmid with an additional RhaS binding site (+RhaS-BS) directly downstream from the annotated *E. coli* −10 region of P*_rhaBAD_*. Additional binding of the RhaS-l-rhamnose complex downstream from the −10 region should potentially contribute to the repression of P*_rhaBAD_*. Also, it was interesting to see the general impact of this additional RhaS BS on the P*_rhaBAD_* activity in the absence of l-rhamnose.

**Figure 4:**
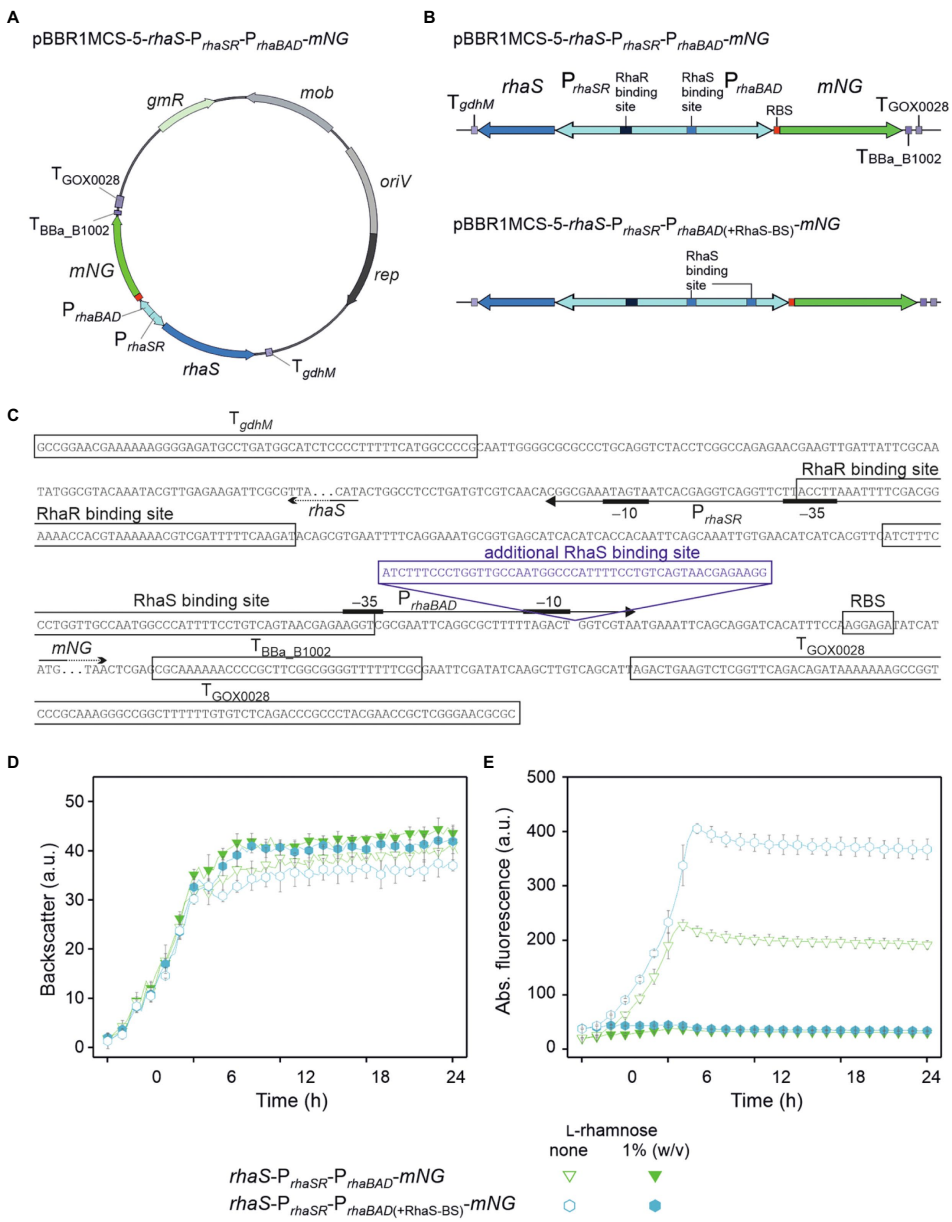
Insertion of an additional RhaS binding site downstream from the −10 region doubled the expression strength of P*_rhaBAD_* and the range of repression. **(A)** Map of plasmid pBBR1MCS-5-*rhaS*-P*_rhaSR_-*P*_rhaBAD_-mNG*. **(B)** Schematic illustration of the pBBR1MCS-5 inserts *rhaS*-P*_rhaSR_-*P*_rhaBAD_*-*mNG* and its variant *rhaS*-P*_rhaSR_-*P_*rhaBAD*(+RhaS-BS)_*-mNG* harboring an additional RhaS binding site directly downstream from the −10 region, all flanked by terminators. **(C)** DNA sequence details of the fragment *rhaS*-P*_rhaSR_*-P_*rhaBAD*(+RhaS-BS)_-*mNG* with RhaS and RhaR binding sites as well as terminator sequences adjacent to *rhaS* and *mNG*. The promoter elements are given according to Egan and Schleif (1993). **(D)** Growth according to backscatter and **(E)** absolute mNG fluorescence of *G. oxydans* 621H carrying either plasmid pBBR1MCS-5-*rhaS*-P*_rhaSR_*-P*_rhaBAD_*-*mNG* or pBBR1MCS-5-*rhaS*-P*_rhaSR_*-P_*rhaBAD*(+RhaS-BS)_-*mNG* in microscale BioLector cultivations in d-mannitol medium without and with 1% (w/v) l-rhamnose. Data represent mean values and standard deviation from two biological replicates (clones) with three technical replicates each. BioLector settings: backscatter gain 20, fluorescence gain 40.

We used plasmid pBBR1MCS-5-*rhaS*-P*_rhaSR_*-P*_rhaBAD_*-*mNG* as template and created a copy of the 50 bp region comprising the native RhaS-BS present in P*_rhaBAD_*. This copy was inserted directly downstream from the −10 region of P*_rhaBAD_*. The resulting plasmid was termed pBBR1MCS-5-*rhaS*-P*_rhaSR_*-P_*rhaBAD*(+RhaS-BS)_-*mNG* and its expression performance was compared with that of the template plasmid (Figure 4). In d-mannitol medium without and with 1% (w/v) l-rhamnose, both strains showed similar growth independent of the plasmids or l-rhamnose supplementation (Figure 4D). Interestingly, in the absence of l-rhamnose, the maximal mNG fluorescence observed for the plasmid carrying +RhaS-BS was almost twice (405 a.u.) that of the parental plasmid (225 a.u.), suggesting additional activation of P*_rhaBAD_* by RhaS in the absence of l-rhamnose or a new transcriptional start increasing the *mNG* expression (Figure 4E). In the presence of 1% (w/v) l-rhamnose, the absolute residual *mNG* expression were similarly low for both constructs according to the mNG fluorescence. Therefore, the relative residual plasmid-based mNG expression was decreased to 11% by +RhaS-BS due to the doubled absolute expression strength in the absence of l-rhamnose (11% for +RhaS-BS: 405 a.u. reduced to 45 a.u.; 17% for parental: 225 a.u. reduced to 38 a.u.).

**Figure 5:**
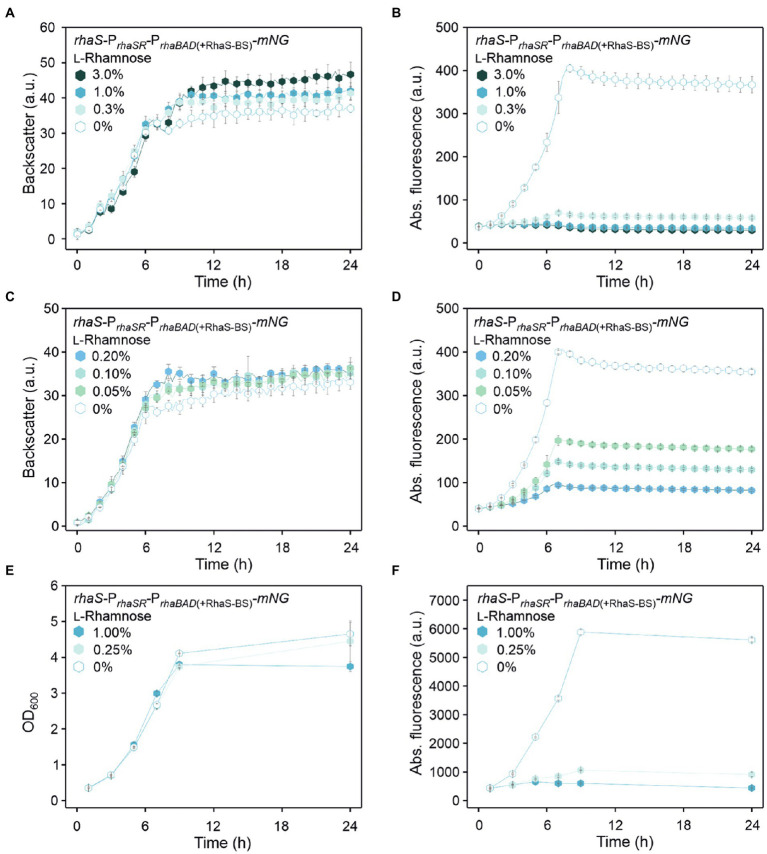
Tunability of the RhaS-P_*rhaBAD*(+RhaS-BS)_ system in *G. oxydans* 621H carrying plasmid pBBR1MCS-5-*rhaS*-P*_rhaSR_*-P_*rhaBAD*(+RhaS-BS)_-*mNG*. **(A,C)** Growth in d-mannitol medium according to backscatter and **(B,D)** absolute mNG fluorescence in BioLector cultivations. l-Rhamnose was supplemented in concentrations ranging from 0.05 to 3% (w/v). All data represent mean values and standard deviation from two biological replicates (clones) with three technical replicates each. BioLector settings: backscatter gain 20, fluorescence gain 40. **(E)** Growth in d-mannitol medium and **(F)** absolute mNG fluorescence in shake flasks. The mNG fluorescence was measured in a Tecan reader (gain 60).

The tunability of P_*rhaBAD*(+RhaS-BS)_ repression was tested with 0.05%, 0.1%, 0.2%, 0.3%, 1%, and 3% (w/v) l-rhamnose (Figures 5A–D). With 1% and 3% (w/v), the reduction of the mNG fluorescence was similarly high (from 405 a.u. to 43 and 41 a.u., respectively), indicating that like P*_rhaBAD_*, plasmid-based P_*rhaBAD*(+RhaS-BS)_ is also almost maximally repressed by 1% (w/v) l-rhamnose. The calculated residual *mNG* expression from P_*rhaBAD*(+RhaS-BS)_ was 11% and 10%, respectively. With 0.3% (w/v) l-rhamnose, the residual mNG fluorescence was 17% (405 vs. 69 a.u.). With only 0.05% (w/v) l-rhamnose, the mNG fluorescence was reduced approximately by half (from 406 to 197 a.u.), showing the sensitivity and tunability of the system. In shake flask cultivations with 0.25% and 1% (w/v) l-rhamnose, P_*rhaBAD*(+RhaS-BS)_ showed a similar repression performance as in microscale BioLector conditions. After 9 h of growth in shake flasks, the maximal mNG fluorescence without l-rhamnose (5,833 a.u.) was reduced to 1,060 and 600 a.u. in the presence of 0.25% and 1% (w/v) l-rhamnose (Figures 5E,F), representing 18% and 10% residual *mNG* expression.

**Figure 6:**
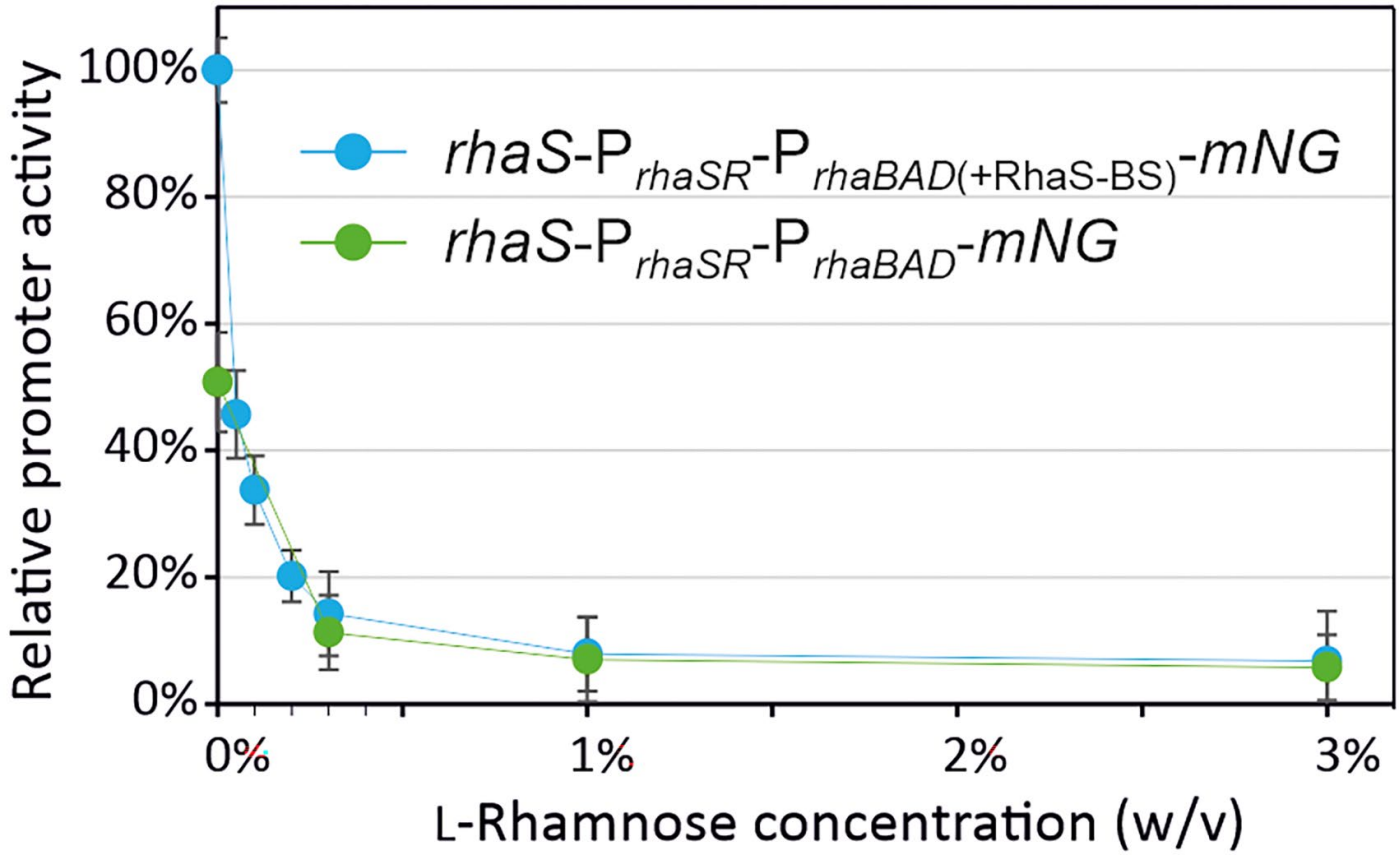
Responsiveness of P*_rhaBAD_* and P_*rhaBAD*(+RhaS-BS)_ towards l-rhamnose. The maximal mNG fluorescence of *G. oxydans* 621H carrying either plasmid pBBR1MCS-5-*rhaS*-P*_rhaSR_*-P*_rhaBAD_*-*mNG* or pBBR1MCS-5-*rhaS*-P*_rhaSR_*-P_*rhaBAD*(+RhaS-BS)_-*mNG* in response to different l-rhamnose concentrations was used to calculate relative % promoter activities compared to the maximal P_*rhaBAD*(+RhaS-BS)_ activity in the absence of l-rhamnose (100%).

Plotting the relative maximal P*_rhaBAD_*- and P_*rhaBAD*(+RhaS-BS)_-derived mNG fluorescence vs. the l-rhamnose concentrations illustrates the responsiveness of both promoters toward low l-rhamnose concentrations (Figure 6). While the absolute repression of both promoters was similar and down to 10% of the maximal individual expression strength, non-repressed P_*rhaBAD*(+RhaS-BS)_ was two-fold stronger than P*_rhaBAD_* and therefore offers a wider dynamic range of expression.

### A genomic single copy of P_*rhaBAD*(+RhaS-BS)_ can be tuned and completely repressed by RhaS and l-rhamnose

**Figure 7:**
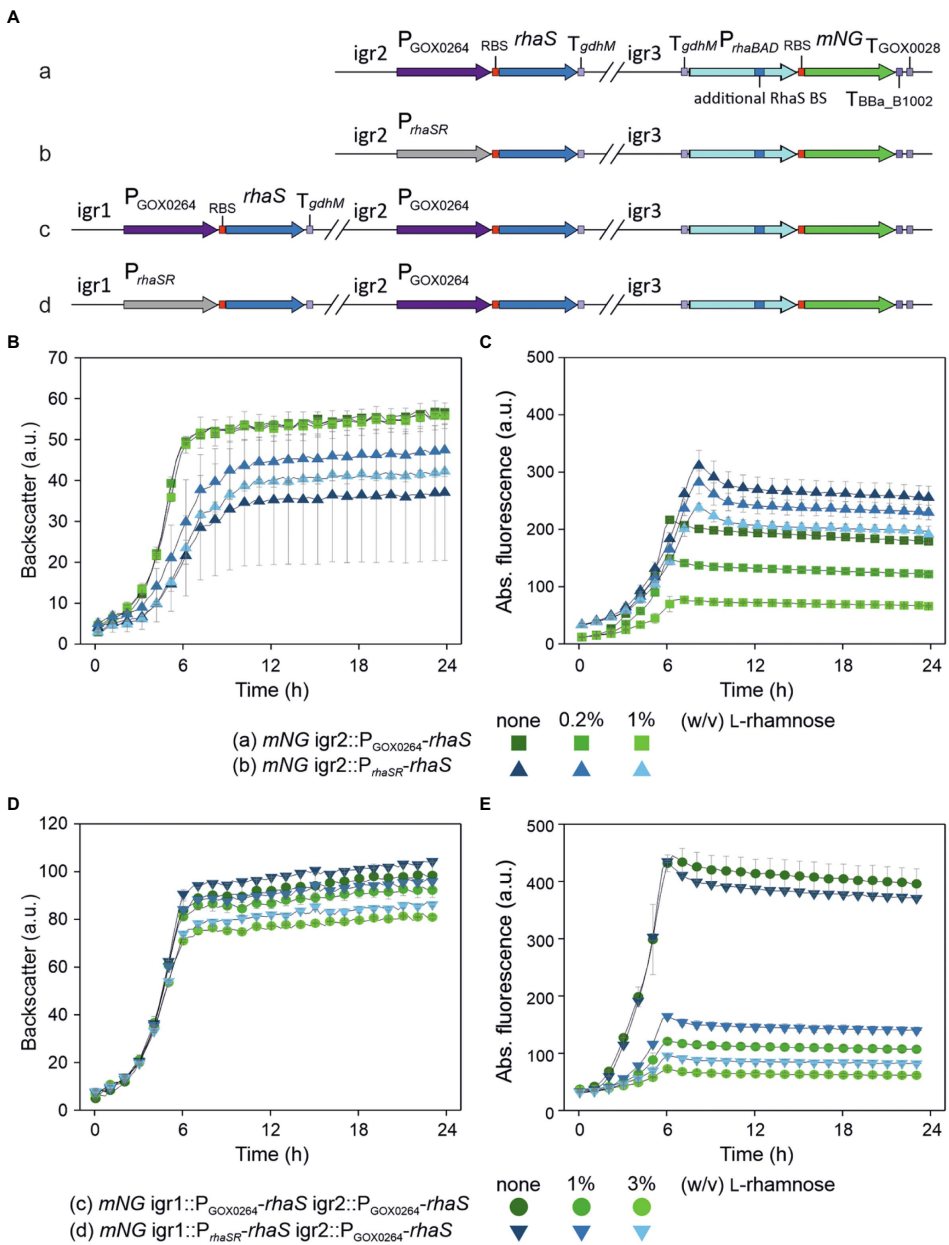
Partial repression of genomic single-copy P_*rhaBAD*(+RhaS-BS)_-*mNG* using genomically integrated copies of *rhaS*. **(A)** Schematic illustration of the genomic backgrounds of the *G. oxydans* 621H strains. The expression cassette P_*rhaBAD*(+RhaS-BS)_-*mNG* of the reporter gene was genomically integrated into the intergenic region igr3 (GOX0038/GOX_RS01330–GOX0039/GOX_RS01335). The resulting strain was termed *G. oxydans mNG*. For single-copy *rhaS* expression, a *rhaS* expression cassette either under control of P_GOX0264_ (a) or P*_rhaSR_* (b) was genomically integrated in *G. oxydans mNG* into igr2 (GOX0028/GOX_RS01280 - GOX0029/GOX_RS01285). A second *rhaS* expression cassette again either under control of P_GOX0264_ (c) or P*_rhaSR_* (d) was genomically integrated into igr1 (GOX0013/GOX_RS01200 - GOX0014/GOX_RS01205) in strain A with P_GOX0264_-*rhaS* in igr2. **(B,D)** Growth of the strains in d-mannitol medium according to backscatter and **(C,E)** absolute mNG fluorescence in BioLector cultivations. l-Rhamnose was supplemented as indicated. All data represent mean values and standard deviation from two biological replicates (clones) with three technical replicates each. BioLector settings: backscatter gain 20, fluorescence gain 70.

We then analyzed if the stronger P*_rhaBAD_* variant can be completely repressed in a plasmid-free strain when this modified target promoter and *rhaS* are genomically integrated and present as a single copy instead of being present on a plasmid with medium copy number (Figure 7A). Therefore, we integrated the reporter gene *mNG* under control of P_*rhaBAD*(+RhaS-BS)_ into the intergenic region igr3 (GOX0038/GOX_RS01330–GOX0039/GOX_RS01335). The resulting strain was termed *G. oxydans mNG*. For single-copy *rhaS* expression, we tested the promoters P*_rhaSR_* and P_GOX0264_ and integrated both *rhaS* constructs in *G. oxydans mNG* separately into igr2 (GOX0028/GOX_RS01280–GOX0029/GOX_RS01285). The resulting *G. oxydans* strains *mNG* igr2::P_GOX0264_-*rhaS* and *mNG* igr2::P*_rhaSR_*-*rhaS* were cultivated and analyzed in a BioLector (Figures 7B,C). As observed before with the plasmid-based approach, in the absence of l-rhamnose expression of single-copy *rhaS* under control of P*_rhaSR_* resulted in higher activity of P_*rhaBAD*(+RhaS-BS)_ than with P_GOX0264_-*rhaS*. However, with *rhaS* under control of P_GOX0264_ a much higher extent of repression was observed with 1% (w/v) l-rhamnose. Here, the maximal mNG signals were reduced by 64% from 217 to 78 a.u. (Figure 7C). These results indicated that single-copy *rhaS* expression is not sufficient to completely repress P_*rhaBAD*(+RhaS-BS)_. We then tested if a second genomic *rhaS* copy could be sufficient and integrated both P_GOX0264_-*rhaS* and P*_rhaSR_*-*rhaS* into strain *mNG* igr2::P_GOX0264_-*rhaS* separately into igr1 (GOX0013/GOX_RS01200–GOX0014/GOX_RS01205). The two resulting *G. oxydans* strains *mNG* igr1::P_GOX0264_-*rhaS* igr2::*rhaS* and *mNG* igr1::P*_rhaSR_*-*rhaS* igr2::*rhaS* were cultivated and analyzed in a BioLector (Figures 7D,E). The extent of repression in the presence of l-rhamnose was higher with two *rhaS* copies compared to only one copy and again P_GOX0264_-*rhaS* performed better in repression than P*_rhaSR_*-*rhaS*, yet two genomic *rhaS* copies were still not sufficient to completely repress P_*rhaBAD*(+RhaS-BS)_. With one copy of P*_rhaSR_*-*rhaS* and one copy of P_GOX0264_-*rhaS* the maximal mNG signals were reduced by 78% from 435 to 96 a.u. With two genomic copies of P_GOX0264_-*rhaS*, the maximal mNG signals were reduced by 84% from 444 to 73 a.u. (Figure 7E).

**Figure 8:**
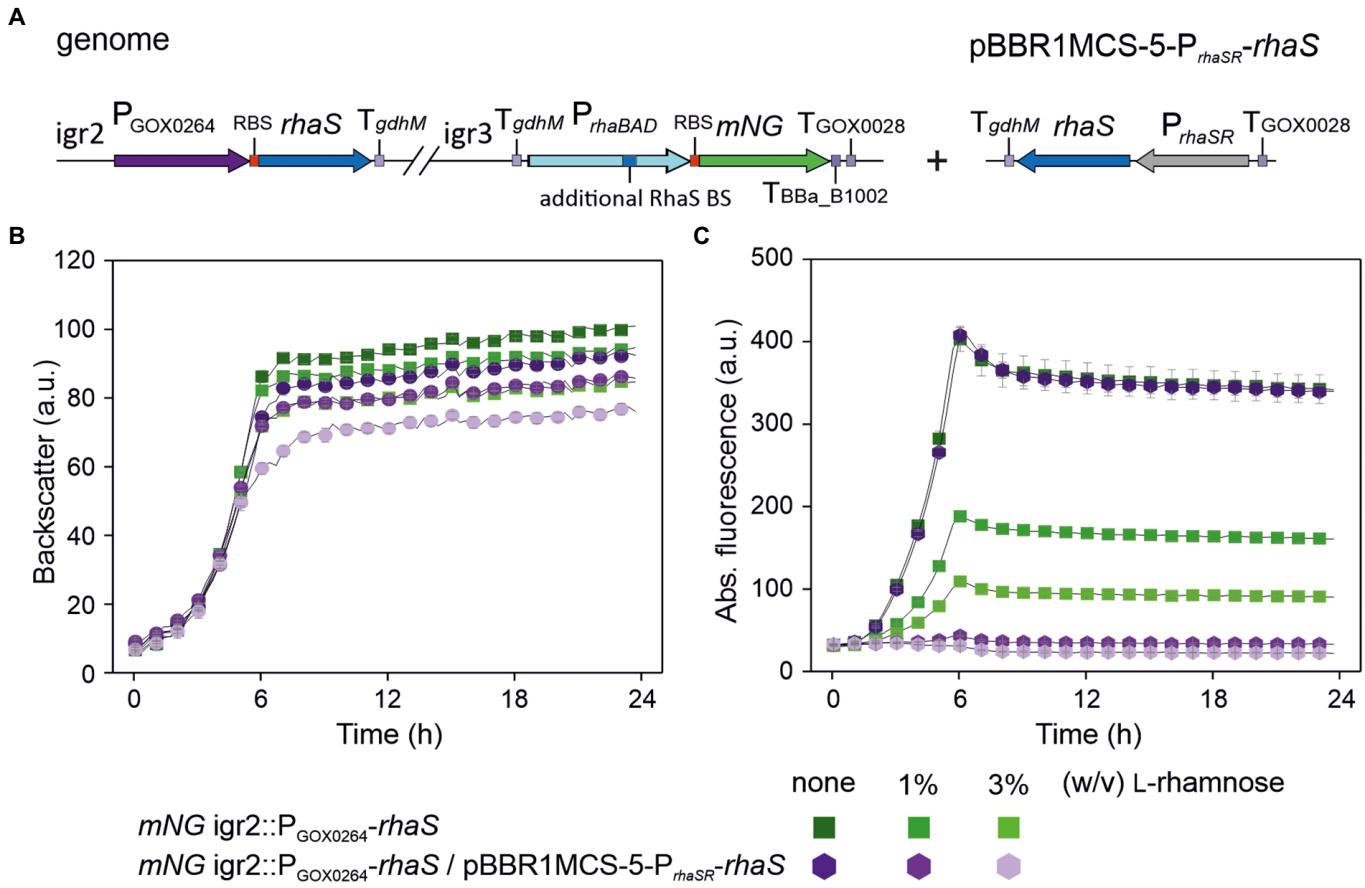
Complete repression of genomic single-copy P_*rhaBAD*(+RhaS-BS)_-*mNG* by plasmid-based expression of *rhaS* and l-rhamnose. **(A)** Schematic illustration of the genomic background as described in Figure 7A (variant a) of the plasmid-carrying *G. oxydans mNG* igr2::P_GOX0264_-*rhaS* strain. **(B)** Growth in d-mannitol medium according to backscatter and **(C)** absolute mNG fluorescence in BioLector cultivations. For comparison, the plasmid-free strain with only the single-copy *rhaS* in igr2 was included in the BioLector run. l-Rhamnose was supplemented as indicated. Data represent mean values and standard deviation from three technical replicates of one clone (*mNG* igr2::P_GOX0264_-*rhaS*/pBBR1-MCS-5-P*_rhaSR_*-*rhaS*) and two clones (*mNG* igr2::P_GOX0264_-*rhaS*). BioLector settings: backscatter gain 20, fluorescence gain 70.

To test if a genomic single-copy P_*rhaBAD*(+RhaS-BS)_ can be completely repressed at all, we constructed the *rhaS* expression plasmid pBBR1MCS-5-P*_rhaSR_*-*rhaS* and introduced it into the single-copy *rhaS* strain *G. oxydans mNG* igr2::P_GOX0264_-*rhaS* already showing 64% promoter repression (Figure 8A). The resulting plasmid-carrying strain was cultivated and analyzed in a BioLector (Figures 8B,C). According to the mNG signals, the genomic single-copy P_*rhaBAD*(+RhaS-BS)_ appeared completely repressed by 3% and possibly also by 1% (w/v) l-rhamnose. To test the tunability of this repression with plasmid-based expression of *rhaS*, we also tested lower l-rhamnose concentrations (Supplementary Figure S5). In the presence of 0.1% (w/v) l-rhamnose, the maximal mNG signals were reduced by 64% from 216 to 78 a.u.. In the presence of 0.2% (w/v) l-rhamnose, the maximal mNG signals were reduced by 78% from 216 to 47 a.u.. These results indicated a relatively high sensitivity of the system toward lower l-rhamnose concentrations and that a genomic copy of the RhaS target promoter variant can be tuned.

### The *Escherichia coli* promoter P*_rhaT_* is weak, inducible and tunable in *Gluconobacter oxydans*

**Figure 9:**
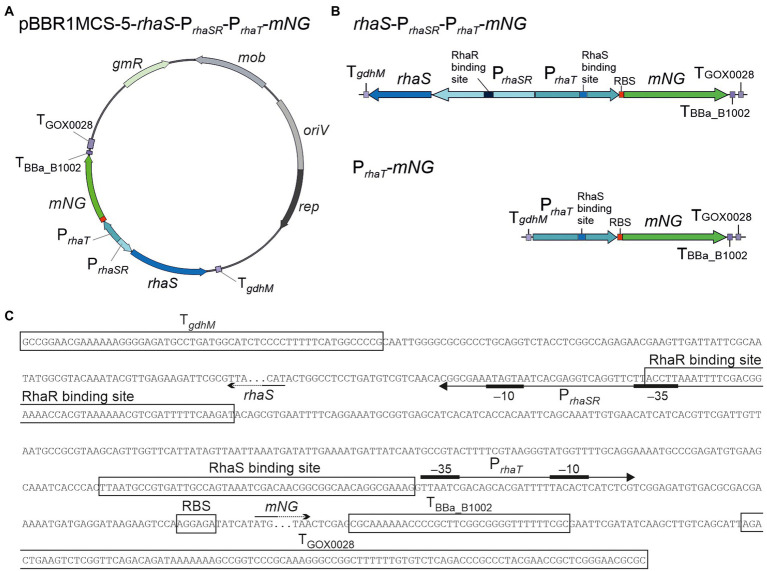
Reporter plasmids with P*_rhaT_* and sequence details. **(A)** Map of plasmid pBBR1MCS-5-*rhaS*-P*_rhaSR_-*P*_rhaT_-mNG* with the fluorescence reporter gene *mNeonGreen* (*mNG*) under control of the promoter P*_rhaT_* from the l-rhamnose transporter gene *rhaT* with the adjacent *rhaS* gene under control of P*_rhaSR_*, all flanked by the terminators T*_gdhM_*, T_BBa_B1002_ and T_GOX0028_. **(B)** Schematic illustration of the pBBR1MCS-5 inserts *rhaS*-P*_rhaSR_-*P*_rhaT_*-*mNG* and its variant P*_rhaT_-mNG* lacking *rhaS*-P*_rhaSR_*. **(C)** DNA sequence details with RhaS and RhaR binding sites and terminator sequences downstream from *rhaS* and *mNG*. P*_rhaT_* promoter elements are given according to Vía et al. (1996).

As mentioned above, in *E. coli* RhaS also activates the promoter P*_rhaT_* of the l-rhamnose transporter gene *rhaT*. Similar to P*_rhaBAD_*, P*_rhaT_* contains two regulatory elements, one for RhaS and one for CRP binding. Contrary to P*_rhaBAD_*, the RhaS binding site on P*_rhaT_* is differently composed and slightly shifted, so that the binding site does not overlap with the −35 element of P*_rhaT_* (Vía et al., 1996; Wickstrum et al., 2010). To analyze the regulation and performance of P*_rhaT_* by RhaS in *G. oxydans*, we constructed reporter plasmid pBBR1MCS-5-*rhaS*-P*_rhaSR_*-P*_rhaT_*-*mNG*. As a control, plasmid pBBR1MCS-5-P*_rhaT_*-*mNG* lacking *rhaS* was constructed (Figure 9).

**Figure 10:**
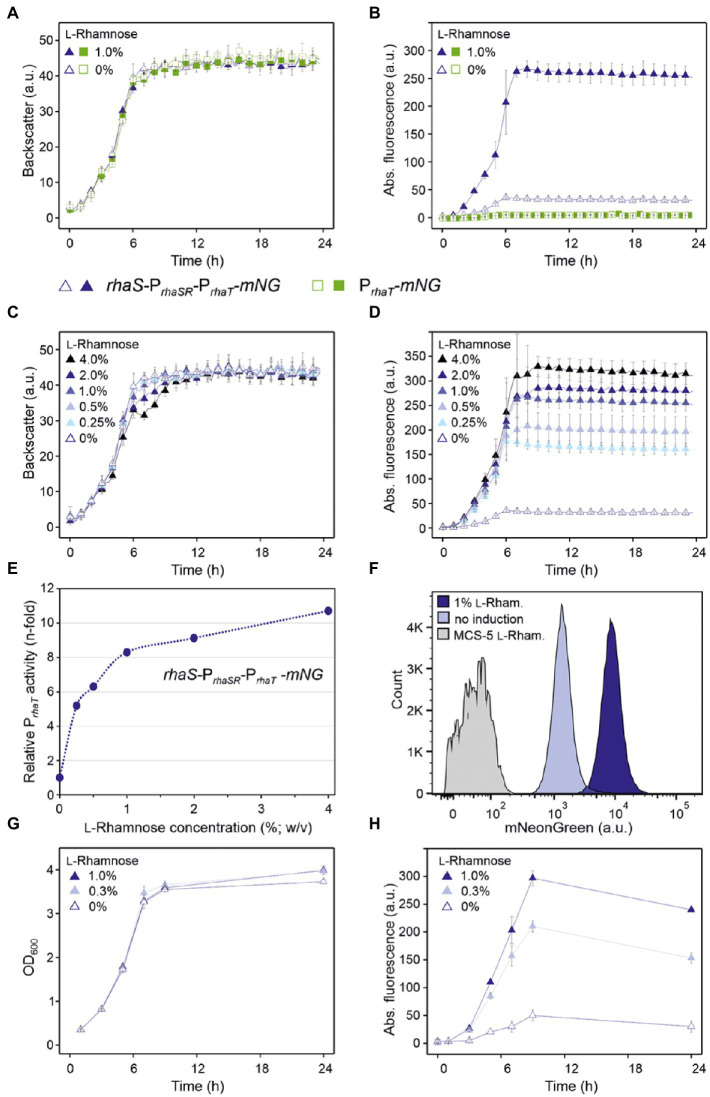
Performance of the RhaS-P*_rhaT_* system in *G. oxydans* 621H. **(A)** Growth according to backscatter and **(B)** absolute mNG fluorescence of *G. oxydans* 621H carrying plasmid pBBR1MCS-5-*rhaS*-P*_rhaSR_*-P*_rhaT_*-*mNG* or pBBR1MCS-5-P*_rhaT_*-*mNG* lacking *rhaS* in microscale BioLector cultivations without and with 1% (w/v) l-rhamnose. **(C)** Growth (backscatter) and **(D)** absolute mNG fluorescence of *G. oxydans* 621H carrying plasmid pBBR1MCS-5-*rhaS*-P*_rhaSR_*-P*_rhaT_-mNG* in microscale BioLector cultivations with l-rhamnose concentrations from 0.25% to 4% (w/v) as indicated. BioLector settings: backscatter gain 20, fluorescence gain 70. **(E)** Correlation between the relative n-fold P*_rhaT_* activity in *G. oxydans* 621H carrying plasmid pBBR1MCS-5-*rhaS*-P*_rhaSR_*-P*_rhaT_*-*mNG* and the l-rhamnose concentrations. For the calculation, the maximal mNG fluorescence in the absence of l-rhamnose was set to 1. **(F)** FACS analysis of *G. oxydans* 621H carrying plasmid pBBR1MCS-5-*rhaS*-P*_rhaSR_*-P*_rhaT_*-*mNG* or empty vector pBBR1MCS-5 (MCS-5) as a control. Cells were grown in shake flasks with d-mannitol medium without and with 1% (w/v) l-rhamnose. FACS analysis was performed 7 h after inoculation (induction). Total counts per sample represent 100,000 events. **(G)** Growth (OD_600_) and **(H)** l-Rhamnose-induced mNG fluorescence of *G. oxydans* 621H carrying plasmid pBBR1MCS-5-*rhaS*-P*_rhaSR_*-P*_rhaT_-mNG* in shake flask cultivations with d-mannitol medium. The mNG fluorescence was measured in a Tecan reader (gain 60). All data represent mean values and standard deviation from two biological replicates (clones) with three technical replicates each.

In BioLector cultivations, *G. oxydans* cells with pBBR1MCS-5-*rhaS*-P*_rhaSR_*-P*_rhaT_*-*mNG* or pBBR1MCS-5-P*_rhaT_*-*mNG* showed very similar growth independent of the presence or absence of l-rhamnose (Figure 10A). Interestingly and in contrast to P*_rhaBAD_*, *mNG* expression controlled by P*_rhaT_* was induced by l-rhamnose. Addition of 1% (w/v) l-rhamnose increased mNG fluorescence ~7.5-fold (from 36 to 266 a.u.) within 8 h. The values indicated a weak or moderate strength of P*_rhaT_* in *G. oxydans* (Figure 10B). Almost no mNG fluorescence was observed in the strain with plasmid pBBR1MCS-5-P*_rhaT_*-*mNG* without *rhaS*. Thus, on the one hand P*_rhaT_* was almost not active in *G. oxydans* without RhaS and an endogenous *G. oxydans* protein did not interfere. On the other hand, RhaS apparently weakly activated P*_rhaT_* already in the absence of l-rhamnose since with plasmid pBBR1MCS-5-*rhaS*-P*_rhaSR_*-P*_rhaT_*-*mNG* a low basal mNG fluorescence was observed also in the absence of l-rhamnose exceeding the extremely low mNG signals when *rhaS* was absent (Figure 10B). Alternatively, a low level of l-rhamnose could be present in the complex medium resulting in a basal RhaS-dependent induction of the system. It should be noted that due to the relatively weak expression from P*_rhaT_* compared to P*_rhaBAD_*, in these BioLector cultivations the fluorescence signals were monitored with gain 70 instead of gain 40 or 50.

The tunability of P*_rhaT_* induction was tested with l-rhamnose concentrations ranging from 0.25% to 4% (w/v). Again, growth of *G. oxydans* cells with pBBR1MCS-5-*rhaS*-P*_rhaSR_*-P*_rhaT_*-*mNG* was largely unaffected by up to 2% (w/v) l-rhamnose (Figure 10C). With 4% (w/v) l-rhamnose, the backscatter data suggested a biphasic growth. The P*_rhaT_*-derived *mNG* expression increased gradually in an inducer-dependent manner (Figure 10D). The maximal induction observed was 9.2-fold (36 vs. 330 a.u.) and required 4% (w/v) l-rhamnose. With 0.25% (w/v) l-rhamnose already half of the maximal induction was reached showing that the weak to moderate P*_rhaT_*-derived *mNG* expression could be nicely tuned by low l-rhamnose concentrations (Figure 10E). The low expression strength of P*_rhaT_* and its tunability could be of particular interest for the synthesis of proteins forming inclusion bodies when expressed at higher levels.

The homogeneity of P*_rhaT_* induction was analyzed by FACS using cells harvested after 7 h of growth in d-mannitol medium without or with 1% (w/v) l-rhamnose (Figure 10F). In the absence of l-rhamnose, 97.4% of the analyzed cells with pBBR1MCS-5-*rhaS*-P*_rhaSR_*-P*_rhaT_*-*mNG* showed relatively low fluorescence signals (~1,000 a.u.). In the presence of 1% (w/v) l-rhamnose, 96.9% of the population showed approximately 9-fold higher mNG fluorescence signals (~9,000 a.u.). We also tested the inducible P*_rhaT_*-derived *mNG* expression in shake flask cultures with 0.3% and 1% (w/v) l-rhamnose. Under these conditions, all cultures with pBBR1MCS-5-*rhaS*-P*_rhaSR_*-P*_rhaT_*-*mNG* exhibited very similar growth (Figure 10G). The *mNG* expression was similarly induced as in the BioLector cultivations (Figure 10H). The maximal mNG fluorescence was reached after 9 h of growth and represented 4-fold and 6-fold induction with 0.3% (50 vs. 210 a.u.) and 1% (w/v) l-rhamnose (50 vs. 297 a.u.), respectively.

**Figure 11:**
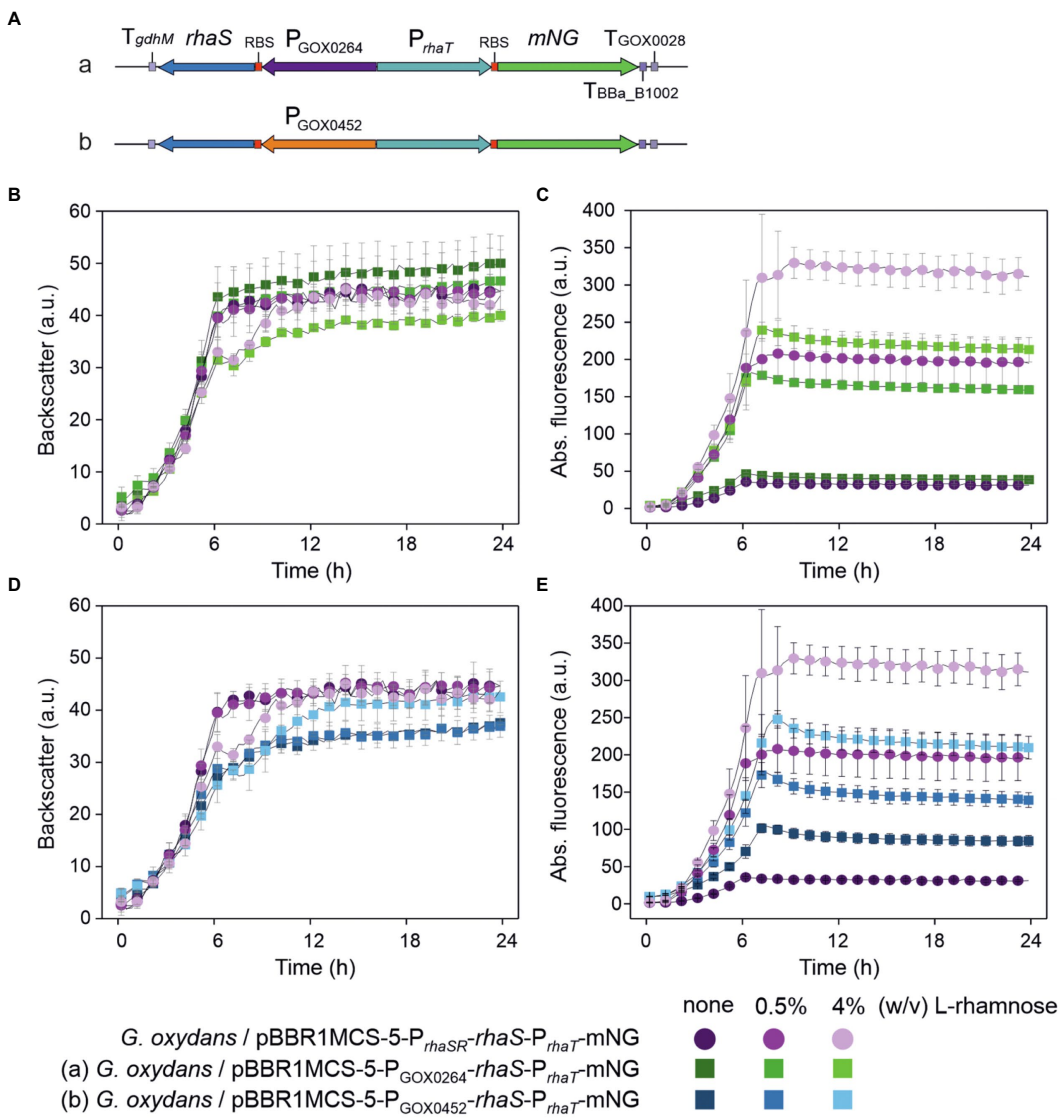
Performance of P*_rhaT_*-derived induction of *mNG* expression in dependence of *rhaS* expression and presence of l-rhamnose. **(A)** Schematic illustration of the pBBR1MCS-5 plasmid inserts to test the effects of *rhaS* expression. **(B,D)** Growth of the *G. oxydans* 621H strains with *rhaS* expression plasmid in d-mannitol medium according to backscatter and **(C,E)** absolute mNG fluorescence in BioLector cultivations. l-Rhamnose was supplemented as indicated. All data represent mean values and standard deviation from two biological replicates (clones) with three technical replicates each. BioLector settings: backscatter gain 20, fluorescence gain 70.

To test the influence of *rhaS* expression strength from different promoters on the performance of the RhaS-P*_rhaT_* system, we replaced P*_rhaSR_* and constructed plasmid variants with P_GOX0264_-*rhaS* and P_GOX0452_-*rhaS* (Figure 11A). The *G. oxydans* strains with either of the reporter plasmids were cultivated and analyzed in a BioLector to compare the basal expression level and the induction performance with that of cells expressing *rhaS* under the control of P*_rhaSR_* (Figures 11B–E). For both tested *G. oxydans* promoters the maximal mNG signals with 4% (w/v) l-rhamnose were ~25% lower compared to that obtained with P*_rhaSR_*-*rhaS*. Since the non-induced maximal mNG signals obtained with P*_rhaT_* were somewhat higher with P_GOX0264_-*rhaS* (46 a.u.) and were approximately 3-fold higher with P_GOX0452_-*rhaS* (104 a.u.) compared to P*_rhaSR_*-*rhaS* (36 a.u.), the maximal induction fold changes with 4% (w/v) l-rhamnose were only 5-fold with P_GOX0264_-*rhaS* and 2.4-fold with P_GOX0452_-*rhaS*. Thus, compared to P*_rhaSR_*-*rhaS* the non-induced basal expression level was not lowered and the induction fold changes of the RhaS-P*_rhaT_* system were not improved by using P_GOX0264_ or P_GOX0452_ for *rhaS* expression.

### Insertion of an additional RhaS binding site can reverse the regulation making P*_rhaT_* repressible by RhaS and l-rhamnose

**Figure 12:**
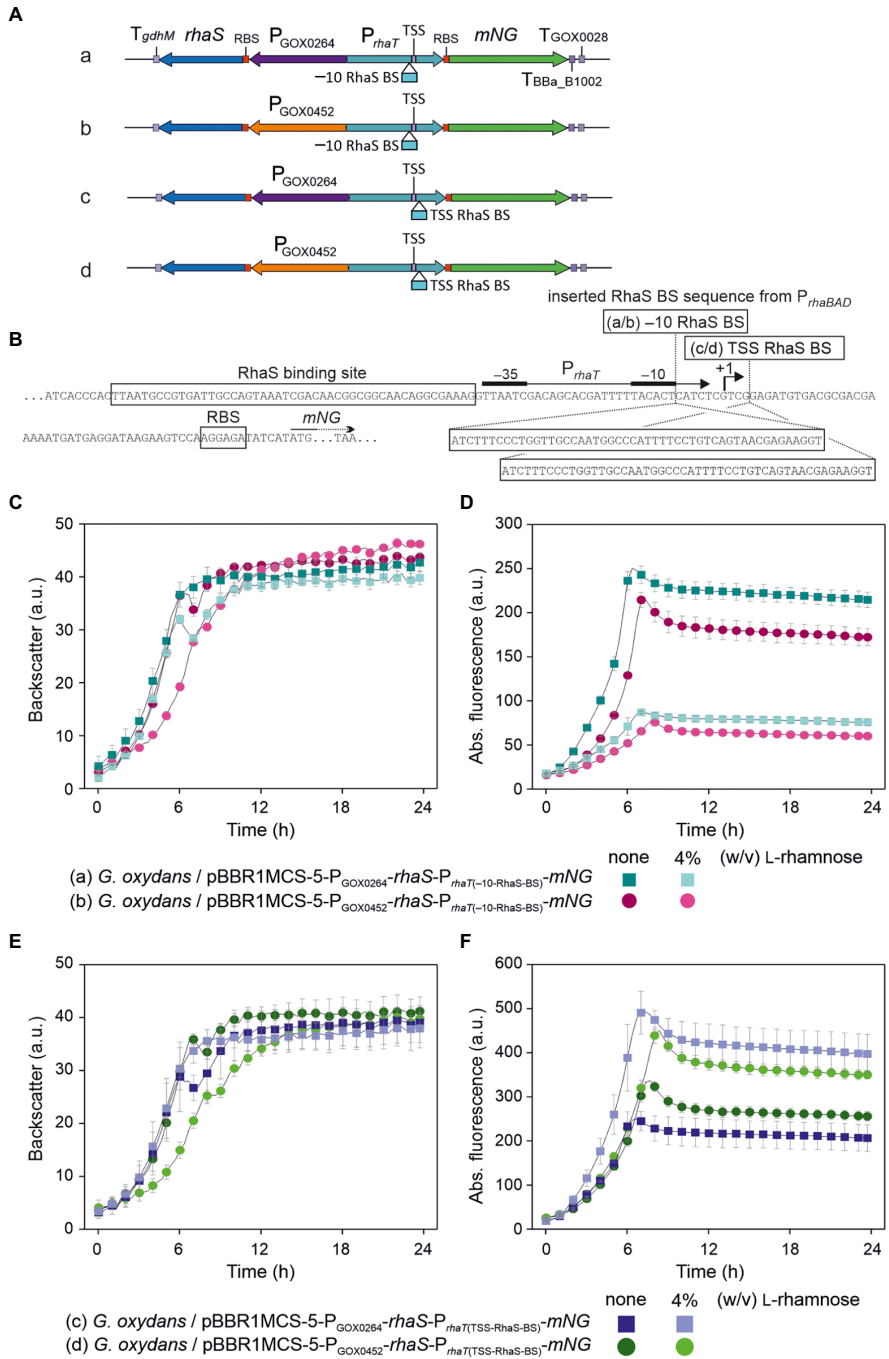
Insertion of an additional RhaS binding site directly downstream from the *E. coli* −10 region of P*_rhaT_* reversed the regulation in *G. oxydans* making the modified RhaS-P*_rhaT_* system repressible in the presence of l-rhamnose. **(A)** Schematic illustration of the pBBR1MCS-5 plasmid inserts to test the effects of an additional RhaS binding site (RhaS BS) in P*_rhaT_* directly downstream from the *E. coli* −10 region (−10 RhaS BS) or downstream from the *E. coli* P*_rhaT_* transcriptional start site (TSS RhaS BS) together with *rhaS* expression from P_GOX0264_ or P_GOX0452_. **(B)** Sequence details of P*_rhaT_* with the positions and RhaS binding site sequence from P*_rhaBAD_* inserted either directly downstream from the *E. coli* −10 region or downstream from the *E. coli* transcriptional start site (TSS +1) according to Vía et al. (1996). **(C,E)** Growth of the *G. oxydans* 621H strains with *rhaS* expression plasmid and modified P*_rhaT_* in d-mannitol medium according to backscatter and **(D,F)** absolute mNG fluorescence in BioLector cultivations. l-Rhamnose was supplemented as indicated. All data represent mean values and standard deviation from two biological replicates (clones) with three technical replicates each. BioLector settings: **(C,E)** backscatter gain 20, **(D)** fluorescence gain 60, **(F)** fluorescence gain 70.

To test the influence of an additional RhaS binding site on the expression performance of P*_rhaT_*, we inserted the RhaS binding site sequence from P*_rhaBAD_* on the one hand directly downstream from the *E. coli* −10 region (−10-RhaS-BS) and on the other hand downstream from the *E. coli* TSS (TSS-RhaS-BS), and constructed for both P*_rhaT_* variants expression plasmids with *rhaS* under control of either P_GOX0264_ or P_GOX0452_ (Figures 12A,B). In case of the −10-RhaS-BS, the regulation was reversed and P_*rhaT*(−10-RhaS-BS)_ was repressible. The maximal mNG signals in the absence of l-rhamnose for both *rhaS* constructs P_GOX0264_-*rhaS* (250 a.u.) and P_GOX0452_-*rhaS* (214 a.u.) were reduced by 65% (87 and 77 a.u.; Figures 12C,D). In contrast, the variant P_*rhaT*(TSS-RhaS-BS)_ was still inducible, yet showed increased and relatively high non-induced mNG signals in the absence of l-rhamnose, which could maximally only be doubled by induction with 4% (w/v) l-rhamnose (Figures 12E,F).

## Discussion

In this study, we found that the promoters P*_rhaBAD_* and P*_rhaT_* together with the transcriptional regulator RhaS, all derived from *E. coli*, exhibit interesting characteristics for the control of gene expression in the AAB *G. oxydans*. These characteristics are affected by the *rhaS* expression strength and additional RhaS binding sites in P*_rhaBAD_* and P*_rhaT_*. With RhaS-P*_rhaBAD_* we found the first system for *G. oxydans* that permits controlled down-regulation in an effector-dependent manner exhibiting tunability and enabling complete repression of a genomically encoded target gene. Furthermore, the regulation of P*_rhaT_* could be reversed from inducible to repressible by inserting an additional RhaS binding site. Altogether, these features provide novel opportunities expanding the genetic toolbox for regulatable gene expression in *G. oxydans* and are possibly also interesting for other AAB.

In *E. coli* the l-rhamnose-induced regulation of P*_rhaBAD_* requires both RhaR and RhaS (Egan and Schleif, 1993, 1994; Kelly et al., 2016). In *G. oxydans*, only RhaS played an effective role for the regulation of the system. In *E. coli*, first RhaR activates expression of the *rhaSR* operon in the presence of l-rhamnose, which is a prerequisite to provide sufficient RhaS levels for the induction of P*_rhaBAD_* by RhaS. This stimulation of *rhaSR* expression by RhaR is proposed to be achieved by bending the P*_rhaSR_* promoter DNA so that P*_rhaSR_*-bound cAMP receptor protein (CRP) can interact with the RNA polymerase (RNAP) and thereby activates transcription of *rhaSR* (Wickstrum et al., 2010). Activation of P*_rhaSR_* by RhaR in such a manner is not possible in *G. oxydans* since CRP is absent. The protein showing the highest similarity to CRP was shown to function as an iron–sulfur cluster-containing FNR-type transcriptional regulator (GOX0974/GOX_RS06010) of genes involved in respiration and redox metabolism (Schweikert et al., 2021). In *G. oxydans*, the presence of RhaR even decreased the RhaS-dependent P*_rhaBAD_* activity (Figure 1). This might be caused by a decreased expression of *rhaSR*, resulting in a lower RhaS level. In *E. coli*, l-rhamnose only affects the RhaR-dependent DNA bending and thereby activates transcription from P*_rhaSR_*, yet the binding of RhaR to its target DNA *per se* was not affected by l-rhamnose (Kolin et al., 2008). RhaS can also bind to the RhaR binding site of P*_rhaSR_* leading to lowered expression of the *rhaSR* operon in *E. coli*, thereby providing a negative feedback loop since the RhaS-dependent DNA bending of P*_rhaSR_* is different from the bending by RhaR and prevents CRP-dependent activation of *rhaSR* expression (Wickstrum et al., 2010). In *G. oxydans*, RhaS alone activated P*_rhaSR_* already in the absence of l-rhamnose and l-rhamnose further stimulated this effect (Supplementary Figures S4A,C). Therefore, in *G. oxydans* RhaR likely binds to P*_rhaSR_* and competes with RhaS, causing an inhibition of P*_rhaSR_* activation by RhaS and consequently lowered the RhaS level, resulting in the lower P*_rhaBAD_* activity. Alternatively, or partially, the data obtained with the constructs omitting only *rhaS* and both *rhaSR* suggested that in *G. oxydans* RhaR could also bind to P*_rhaBAD_* and competes with RhaS in binding to P*_rhaBAD_*, resulting in the lowered reporter signals in the absence of l-rhamnose (Figure 1). Hence, omitting *rhaR* and using only *rhaS* provides advantages when using these regulatable *E. coli* promoters for gene expression in *G. oxydans*.

A surprising outcome of this study was the reversed regulation of P*_rhaBAD_* by RhaS in *G. oxydans*, while P*_rhaT_* was still inducible as in *E. coli*. RhaS belongs to the AraC/XylS family of transcriptional regulators (Tobin and Schleif, 1987). Within this protein family most members interact with the C-terminal domain (CTD) of the α-subunit of the RNAP to activate transcription (reviewed in Ebright and Busby, 1995). It was shown that deletion of the RNAP α-CTD reduced expression 180-fold, suggesting a direct interaction of RhaS and the α-CTD of the *E. coli* RNAP (Holcroft and Egan, 2000). Nevertheless, some members of the AraC/XylS family may also activate transcription through interaction with the sigma 70 factor (σ^70^) subunit RpoD of the RNAP. This mode of activation is often indicated by regulator binding sites overlapping with the −35 element of the target promoter (Lonetto et al., 1998; Bhende and Egan, 2000). Within P*_rhaBAD_*, 4 bp of the RhaS binding site overlap with the −35 hexamer of this promoter (Figure 4), while within P*_rhaT_* the RhaS binding site does not overlap and ends 1 bp upstream from the −35 element (Figure 9). Among the family of σ^70^ transcription factors, the C-terminus is highly conserved as it contains DNA-binding domains and well-defined functional regions (Hakimi et al., 2000; Paget, 2015). In alanine substitution experiments, it was shown that D241 and D250 of RhaS and K593 and R599 of σ^70^ are likely interacting residues required for RhaS-dependent activation of P*_rhaBAD_* in *E. coli* (Bhende and Egan, 2000; Wickstrum and Egan, 2004). While the entire σ^70^ amino acid sequences from *G. oxydans* and from *E. coli* K12 exhibit only 49% identity, primarily due to little similarities in the N-terminal part, the C-terminal regions share 84% identity. In the two regions likely involved in −10 and −35 recognition, only two residues are different (Supplementary Figure S6). R448 and R599 in σ^70^ from *E. coli* correspond to K486 and K637 in σ^70^ from *G. oxydans*. R599 is involved in the recognition of the −35 hexamer and in interaction with RhaS in *E. coli* (Bhende and Egan, 2000; Wickstrum and Egan, 2004). Although the exchange is conservative, K637 might contribute to the reversed responsiveness in *G. oxydans*.

As mentioned above, in P*_rhaBAD_* the RhaS binding site overlaps with most of the −35 region by 4 bp while in P*_rhaT_* the RhaS binding site does not overlap with the −35 region and ends 1 bp upstream (Vía et al., 1996). These different distances in DNA binding positions result in different radial orientations of RhaS toward σ^70^-RNAP along the longitudinal DNA axis. Theoretically, with a turn of 36° per bp, a distance of 5 bp turns the radial orientation by 180°, putting RhaS (or σ^70^-RNAP) to the other side of the DNA strand when comparing the theoretical binding of RhaS and σ^70^-RNAP to P*_rhaBAD_* with the binding to P*_rhaT_*. Because of this theoretical difference in the orientation of RhaS toward σ^70^-RNAP, RhaS possibly interacts with the α-CTD of the RNAP in the case of P*_rhaT_* and with σ^70^ in the case of P*_rhaBAD_*. Since the α-CTD and σ^70^ from *G. oxydans* and *E. coli* differ to some extent, the conformational changes of RhaS induced by the binding of l-rhamnose may affect the interactions of RhaS with the α-CTD and with σ^70^ from *G. oxydans* differently compared to the interactions with the α-CTD and with σ^70^ from *E. coli*, finally resulting in the different modes of the regulation of P*_rhaBAD_* and P*_rhaT_* in *G. oxydans*. Interestingly, in the case of P*_rhaSR_*, the RhaR binding site also overlaps with the −35 region as the RhaS binding site in P*_rhaBAD_*. Moreover, one of the major groove regions of each RhaR half site on P*_rhaSR_* is nearly identical to the corresponding half site for RhaS binding on P*_rhaBAD_* and RhaS can also bind to the RhaR binding site in P*_rhaSR_* as mentioned above (Egan and Schleif, 1994). Despite these similarities between P*_rhaSR_* and P*_rhaBAD_*, in contrast to P*_rhaBAD_*, P*_rhaSR_* was still inducible by RhaS and l-rhamnose in *G. oxydans*. These differences in *G. oxydans* cannot be explained without further experimental data. For example, the recognition by and the affinity of σ^70^ to potential −35 and −10 regions in the absence and in the presence of RhaS and therefore the positional binding of the host RNAP to the *E. coli* promoter DNA relative to the RhaS binding position might differ in *G. oxydans* because of different DNA sequence specificities of σ^70^. Therefore, knowledge about the transcriptional starts sites (TSSs) within the three *E. coli* promoter regions P*_rhaBAD_*, P*_rhaT_*, and P*_rhaSR_* in *G. oxydans* is required to better explain the effects, including the activation of P*_rhaBAD_* by RhaS in the absence of l-rhamnose, the repression, and the effects of the additional RhaS binding site inserted into P*_rhaBAD_* and P*_rhaT_*.

In a first and preliminary attempt to obtain such TSS data, we prepared a total RNA sample from *G. oxydans* 621H with plasmid pBBR1MCS-5-*rhaS*-P*_rhaSR_*-P_*rhaBAD*(+RhaS-BS)_-*mNG* cultivated in the complex medium with d-mannitol in the absence of l-rhamnose and harvested in the mid-exponential phase. The RNA sample was sent to Vertis Biotechnologie AG for sample processing and Illumina sequencing to obtain high-quality TSS data (see Materials and Methods). The resulting fastq file comprised 10,255,084 reads (75 bp). After reads trimming and quality filtering, 1,023,259 reads mapped to the sequence of pBBR1MCS-5-*rhaS*-P*_rhaSR_*-P_*rhaBAD*(+RhaS-BS)_-*mNG*. The overall reads mapping showed three prominent reads stacks indicating the three most active transcriptional starts on the plasmid (Supplementary Figure S7). The by far highest stack (~560,000 coverage) corresponded to the annotated promoter region of *gmR* (*aacC1*) conferring gentamycin resistance and was oriented toward *gmR*. The second-highest stack (~175,000 coverage) was upstream from *rhaS* and oriented toward *rhaS*. In contrast to the expectation for *rhaS*, the start position of this stack was not within P*_rhaSR_*, but upstream from P*_rhaSR_* within the P*_rhaBAD_* region between its −35 and −10 regions from *E. coli*. The third-highest stack (~65,000 coverage) was found within the coding region of *rhaS* and oriented toward the 3′ end of *rhaS*. For P*_rhaBAD_*, the insertion of an additional RhaS binding site could possibly generate an additional transcriptional start site in *G. oxydans* enabling the two-fold increased mNG signals described above for P_*rhaBAD*(+RhaS-BS)_. However, in contrast to the expectations, no one or two major TSSs with high coverage toward *mNG* corresponding to the reported *E. coli* TSS and a potential new TSS could be seen. Instead, the detailed reads mapping showed several reads stacks of only medium coverage, partially with scattering start positions, in the P_*rhaBAD*(+RhaS-BS)_ region and the 5′ region of *mNG* (Supplementary Figure S8). Therefore, the mapping data surprisingly suggested several TSSs in this promoter region oriented toward the 3′ end of the reporter gene: 2 or more potential TSSs upstream from *mNG* and 3 or more potential TSSs in the 5′ region of *mNG*. The most upstream potential TSS for *mNG* was very close to the *E. coli* −35 region of P*_rhaBAD_*. These unexpected preliminary results require further and more detailed analysis as well as some comparisons, including the analysis of RNA samples from *G. oxydans* grown in the presence of l-rhamnose, from cells without *rhaS*, and with the other promoters P*_rhaSR_* and P*_rhaT_*.

Summing up and looking ahead, in *G. oxydans* the RhaS-dependent regulation of the *E. coli* RhaS target promoters and variants thereof provide new modes for regulatable gene expression in this AAB and possibly also in other AAB species. Inducible and repressible gene expression in response to l-rhamnose could be achieved simultaneously, which may be especially advantageous for combinatorial engineering. Tunability and complete repression of a genomic promoter copy was tested and shown only with the variant P_*rhaBAD*(+RhaS-BS)_, yet it is likely that also with P*_rhaBAD_* and P_*rhaT*(−10-RhaS-BS)_ complete repression of a genomic copy could be achieved. These promoters cover different ranges of expression strength, which could be selected according to the requirements of the genomic target gene. Tunable and complete promoter repression is also useful for the functional study of essential genes that cannot be deleted. Optimizing genomic *rhaS* expression or further increasing the genomic *rhaS* copy number beyond two to achieve a sufficient RhaS level may finally overcome the necessity of plasmid-based *rhaS* expression to achieve complete chromosomal promoter repression. Furthermore, more TSS data sets and deeper analysis are required to better understand the regulations of the target promoters by RhaS in *G. oxydans*. The TSS results also suggested to analyze the TSSs of heterologous promoters when they are transferred and used in *G. oxydans* or AAB in general. It can be expected that TSS data sets will help to better understand and overcome the difficulties in getting transferred heterologous regulatable expression systems functional and high-performant in AAB.

## Data availability statement

The raw data supporting the conclusions of this article will be made available by the authors, without undue reservation. The Illumina sequencing data are available in the NCBI sequence read archive via the accession numbers PRJNA854345 and PRJNA854679.

## Author contributions

TP and PF designed and supervised the study. PF, MLG, MM, and MH carried out cloning and experiments. JG performed the GC-TOF analysis. PF, MLG, MM, MH, and TP performed data analysis. PF and TP wrote the manuscript. All authors contributed to the article and approved the submitted version.

## Funding

We are grateful to the Federal Ministry of Education and Research (BMBF) for financial support of the project IMPRES (031B0370B). The funding organization did not influence the design of the study or collection, analysis, and interpretation of data, or writing the manuscript.

## Conflict of interest

The authors declare that the research was conducted in the absence of any commercial or financial relationships that could be construed as a potential conflict of interest.

## Publisher’s note

All claims expressed in this article are solely those of the authors and do not necessarily represent those of their affiliated organizations, or those of the publisher, the editors and the reviewers. Any product that may be evaluated in this article, or claim that may be made by its manufacturer, is not guaranteed or endorsed by the publisher.
