# Supplementary material for: The l-rhamnose-dependent regulator RhaS and its target promoters from Escherichia coli expand the genetic toolkit for regulatable gene expression in the acetic acid bacterium Gluconobacter oxydans
Source: Front Microbiol. 2022 Aug 16;13:981767. doi: 10.3389/fmicb.2022.981767 (PMC9429829; doi:10.3389/fmicb.2022.981767)
Supplement: Supplementary file 1 [file Data_Sheet_1.PDF]

The L-rhamnose-dependent regulator RhaS and its target promoters from *Escherichia coli* expand the genetic toolkit for regulatable gene expression in the acetic acid bacterium *Gluconobacter oxydans*

Philipp Moritz Fricke, Mandy Lynn Gries, Maurice Mürköster, Marvin Höninger, Jochem Gätgens, Michael Bott, and Tino Polen*

Forschungszentrum Jülich GmbH, IBG-1: Biotechnology, Institute of Bio- and Geosciences 52425 Jülich, Germany

*** for correspondence:** Dr. Tino Polen t.polen@fz-juelich.de
Phone +49 2461 61 6205

Supplementary Data

Table S1 DNA oligonucleotides used in this study
Table S2 GC-TOF MS results of L-rhamnose biotransformation assays with *G. oxydans*

Figure S1 Scheme of the RhaSR system from *Escherichia coli*
Figure S2 GC-TOF MS results of L-rhamnose biotransformation assays with *G. oxydans*
Figure S3 Growth of *G. oxydans* 621H in shake flasks without and with 1% (w/v) L-rhamnose
Figure S4 P_{rhaSR}-derived mNG expression
Figure S5 Tunable repression of genomic single-copy $P_{rhaBAD(+RhaS-BS)}$
Figure S6 Sequence alignment of σ^{70} from *G. oxydans* 621H and *E. coli* K12
Figure S7 Reads mapping overview for pBBR1MCS-5-*rhaS*-P_{rhaSR}-$P_{rhaBAD(+RhaS-BS)}$-*mNG*
Figure S8 Reads mapping of the P_{rhaSR}-$P_{rhaBAD(+RhaS-BS)}$ promoter region

Table S1 DNA oligonucleotides used in this study

Name	DNA sequence 5' > 3'
PF1	GGGGCGCGCCCTGCAGGTCTAGATTAATCTTTCTGCGAATTGAGATGACG
PF2	ATTGTCCTCTTCACCTTTAGACACCATATGATATCTCCTTGGAAATGTGATCCTGCTGAATTTCATTAC
PF3	CCAAGGAGATATCATATGGTGTCTAAAGGT
PF4	GAGACTTCAGTCTAATGCTGACAAGCTTGATATCGAATTCGCGAAAAAACCCCGCCGAAG
PF5	AATTGGGGCGCGCCCTGCAGGTCTACCTCGGCCAGAGAACGAAG
PF6	ATGACCGTATTCTTTCTGCAATAACGCGAATCTTCTCAACGTATTTG
PF7	AAGATTCGCGTTATTGCAGAAAGAATACGGTCATACTGGCCTCCTGATG
PF8	AATTGGGGCGCGCCCTGCAGGTCTAGAACTGGCCTCCTGATGTCGTCAAC
PF9	TCCAAGGAGATATCATATGACCGTATTACATAGTGTGGATTTTTTTCCG
PF10	TGTAATACGGTCATATGATATCTCCTTGGATTCGGTCTCCCTCGCC
PF11	TCCTGAAAATTCACGCTGTAGTTGCGCCTGAATGAGAGG
PF12	CAGGCGCAACTACAGCGTGAATTTTCAGGAAATGCGGTG
PF13	TAATACGGTCATATGATATCTCCTTGGATAGTGACATTCCAGCTTGGGGC
PF14	CCTGAAAATTCACGCTGTGGCTTCGTGGTGAACGC
PF15	CACCACGAAGCCACAGCGTGAATTTTCAGGAAATGCGGTG
PF16	CCCCGCAATTGGGGCGCGCCCTGCAGGTCTAGAGCGAAAAAACCCCGCCG
PF17	TCAGGCGCAACGAACGTGATGATGTTCACAATTTGCTGAATTG
PF18	TTGTGAACATCATCACGTTCGTTGCGCCTGAATGAGAGG
PF19	TAATGCTGACAAGCTTGATATCGAATTCTTATTGCAGAAAGCCATCCCG
PF20	GTTCACCACGAAGCCGAACGTGATGATGTTCACAATTTGCT
PF21	TTGTGAACATCATCACGTTCGGCTTCGTGGTGAACGC
PF22	CATCAGGAGGCCAGTTCCAAGGAGATATCATATGGTGTCTAAAGGTG
PF23	TGATATCTCCTTGGAACTGGCCTCCTGATGTCG
PF24	GCTGACAAGCTTGATATCGAATTCAATGTGATCCTGCTGAATTTCATTAC
PF25	TCCTGTCAGTAACGAGAAGGTCGCGAATTCAGGCGCTTTTTAGACTATCTTTCCCTGGTTGCCAATGGCCCATTTTCCTGTCAGTAACGAGAAGGTGGTCGTAATGAAATTCAGCAGG
PF26	GGCCCCGCAATTGGGGCGCGCCCTGCAGGTCTAGAGATTGTTAATGCCGCGTAAGCAG
PF27	TTGTCCTCTTCACCTTTAGACACCATATGATATCTCCTTGGACTTCTTATCCTCATCATTTTTCGTCGCG
PF28	CCAAGGAGATATCATATGGTGTCTAAAGGT
PF29	GCGGCATTAACAATCGAACGTGATGATGTTCACAATTTGCTGAATTG
PF30	ATTGTGAACATCATCACGTTCGATTGTTAATGCCGCGTAAGCAGTTG
PF31	CAGCTATGACATGATTACGAATTCGAGCTCGGTACCATCTGGCCGCC
PF32	GGAGATGCCATCAGGCATCTCCCCTTTTTTCGTTCCGGCGCACTAATCCGAAAAGAGCGGTTGTAG
PF33	TCACGACGTTGTAAAACGACGGCCAGTGCCATTACGACATGGAACCGGGC
PF34	CGCTCGGGAACGCGCCTCGATCGATCAGACCTGTGTGTTCATG
PF35	AAGGGGAGATGCCTGATGGCATCTCCCCTTTTTCATGGCCCCGACTGGCCTCCTGATGTCG
PF36	CACAGGTCTGATCGATCGAGGCGCGTTCCCGAG
PF37	TCATTCAGGCGCAACGATTGTTAATGCCGCGTAAG
PF38	GCGGCATTAACAATCGGCTTCGTGGTGAACGC
PF39	GTTCACCACGAAGCCGATTGTTAATGCCGCGTAAGC
PF40	TGACATGATTACGAATTCGAGCTCGGTACGAATTCGAGCTCGGTACTCTC
PF41	TCCTCTCATTCAGGCGCAACAGACAGATAAAAAAAGCCGGTCC
PF42	TCCCCTTTTTTCGTTCCGGCGAACCGAGACTTCAGTCTGCC
PF43	ACGACGGCCAGTGCCAAGCTCACGTCATCATGAAAGTGCATCAC
PF44	GCAGACTGAAGTCTCGGTTCGCCGGAACGAAAAAAGG
PF45	CCGGCTTTTTTTATCTGTCTGTTGCGCCTGAATGAGAGGAAAG
PF46	TTGTGAACATCATCACGTTCCAGACAGATAAAAAAAGCCGGTC
PF47	CGGCTTTTTTTATCTGTCTGGAACGTGATGATGTTCACAATTTGCT
MH3	TCCTCTCATTCAGGCGCAACCCTCGTGAACTGAAAGACCGGGCAGG
MH5	ACAGGGCAGGAGCGAGATATGCCGGAACGAAAAAAGG

MH6	TCCCCTTTTTTCGTTCCGGCATATCTCGCTCCTGCCCTGTG
MH7	TGTGAACATCATCACGTTCCCCTCGTGAACTGAAAGACCGGGCAGG
MH8	GGTCTTTCAGTTCACGAGGGGAACGTGATGATGTTCACAATTTGCT
MH9	ACGACGTTGTAAAACGACGGCCAGTGCCATTACGCTTATGCGTTCGCGCC
MH10	TACGAATTCGAGCTCGGTACAACCTGACCAGCTCAACACTGGG
MM13	CCAATGGCCCATTTTCCTGTCAGTAACGAGAAGGTCATCTCGTCGGAGATGTGACGCGACG
MM14	ACAGGAAAATGGGCCATTGGCAACCAGGGAAAGATAGTGTAAAAATCGTGCTGTCGATTAACCTTTCGC
MM15	CCAATGGCCCATTTTCCTGTCAGTAACGAGAAGGTGAGATGTGACGCGACGAAAAATGATGAGGATAAG
MM16	ACAGGAAAATGGGCCATTGGCAACCAGGGAAAGATCGACGAGATGAGTGTAAAAATCGTGCTGTCGATTAAC

Table S2 GC-TOF-MS results of L-rhamnose biotransformation assays with *G. oxydans* 621H and control samples. Cell suspensions with an OD_{600} of 1.3 were incubated for 24 h at 30°C and 200 rpm in biotransformation buffer supplemented with 2% (w/v) L-rhamnose. As controls, shake flasks either with L-rhamnose without cells, or with cells but without L-rhamnose were used. For GC-TOF-MS analysis cell-free supernatant was prepared. The peaks at GC retention time R_t 12.69 min, 13.55 min, and 13.65 min corresponded to L-rhamnose. The peak areas indicated that L-rhamnose was hardly consumed or converted by strain 621H within 24 h. Furthermore, in the GC-TOF-MS no new peak could be found which could potentially represent an oxidation product of L-rhamnose.

R_t (min)	Area: 0% L-Rham., with cells, t=0	0% L-Rham., with cells, t=24	2% L-Rham., without cells, t=0	2% L-Rham., with cells, t=0	2% L-Rham., with cells, t=24
12.69	9.22E+05	3.48E+05	8.02E+08	6.44E+08	8.23E+08
13.35	-	-	2.94E+08	1.93E+08	3.15E+08
13.55	3.24E+06	1.21E+06	9.49E+08	1.17E+09	9.96E+08
13.65	-	-	5.29E+08	3.65E+08	6.37E+08
Sum:	4.16E+06	1.56E+06	2.57E+09	2.37E+09	2.77E+09

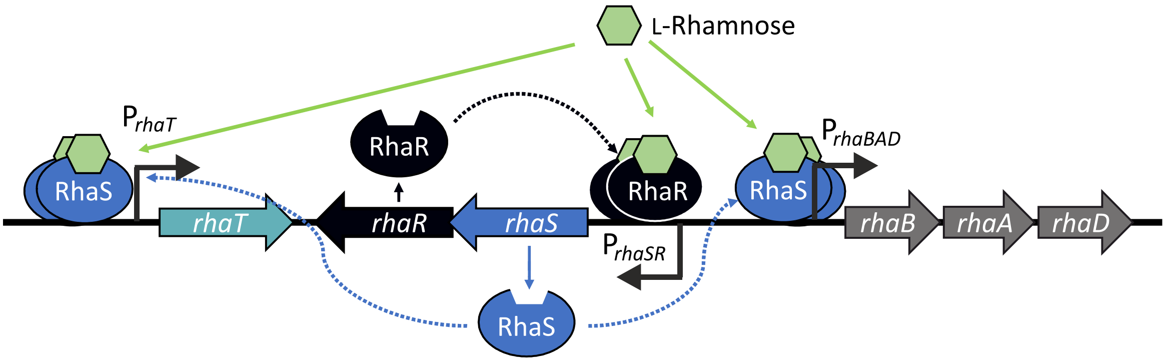

Figure S1 Scheme of the RhaSR system from *Escherichia coli* and positive regulation of the target promotors P_{rhaBAD} and P_{rhaT} by RhaS, and of P_{rhaSR} by RhaR in the presence of L-rhamnose in *E. coli* (modified from Egan and Schleif, 1993;Via et al., 1996).

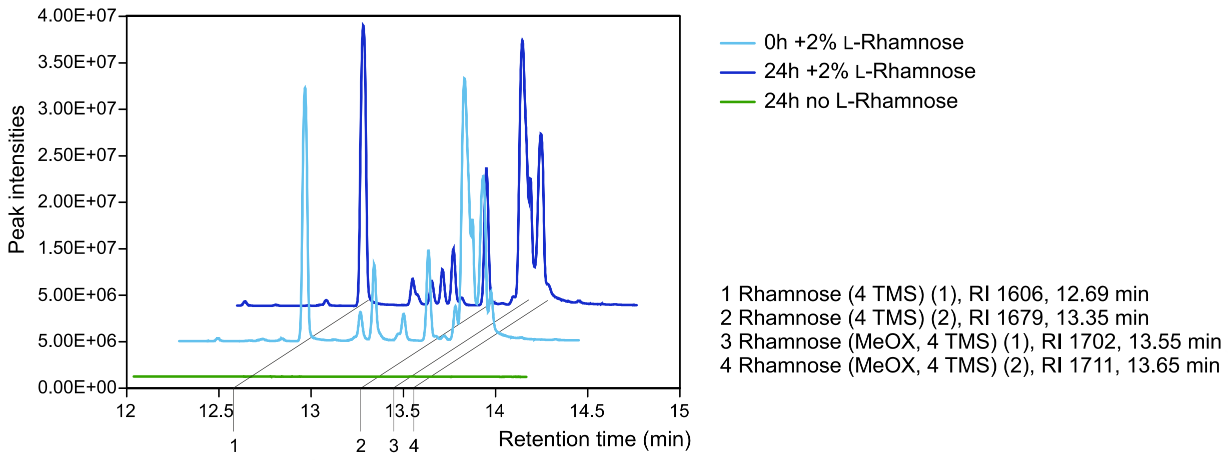

Figure S2 Stacked chromatograms of GC-TOF-MS analysis to check L-rhamnose oxidation by *G. oxydans* 621H in biotransformation assays. Cell suspensions with an OD_{600} of 1.3 were incubated for 24 h at 30°C and 200 rpm in biotransformation buffer supplemented with 2% (w/v) L-rhamnose. Then, cell-free culture supernatant was prepared for GC-TOF-MS analysis.

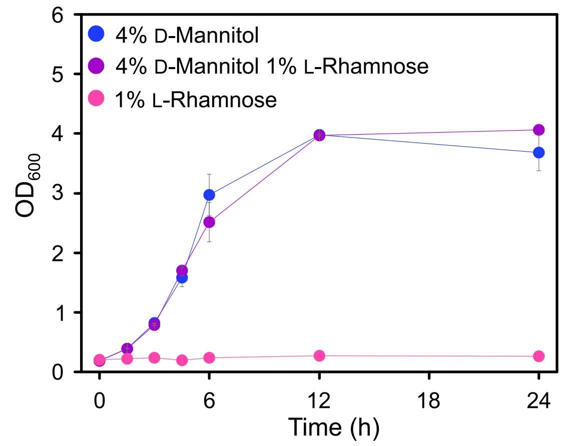

Figure S3 Growth of *G. oxydans* 621H in shake flasks in complex medium with 4% (w/v) D-mannitol supplemented with 1% (w/v) L-rhamnose or not, and in complex medium containing 1% (w/v) L-rhamnose instead of 4% (w/v) D-mannitol. For each condition the data represent two biological replicates.

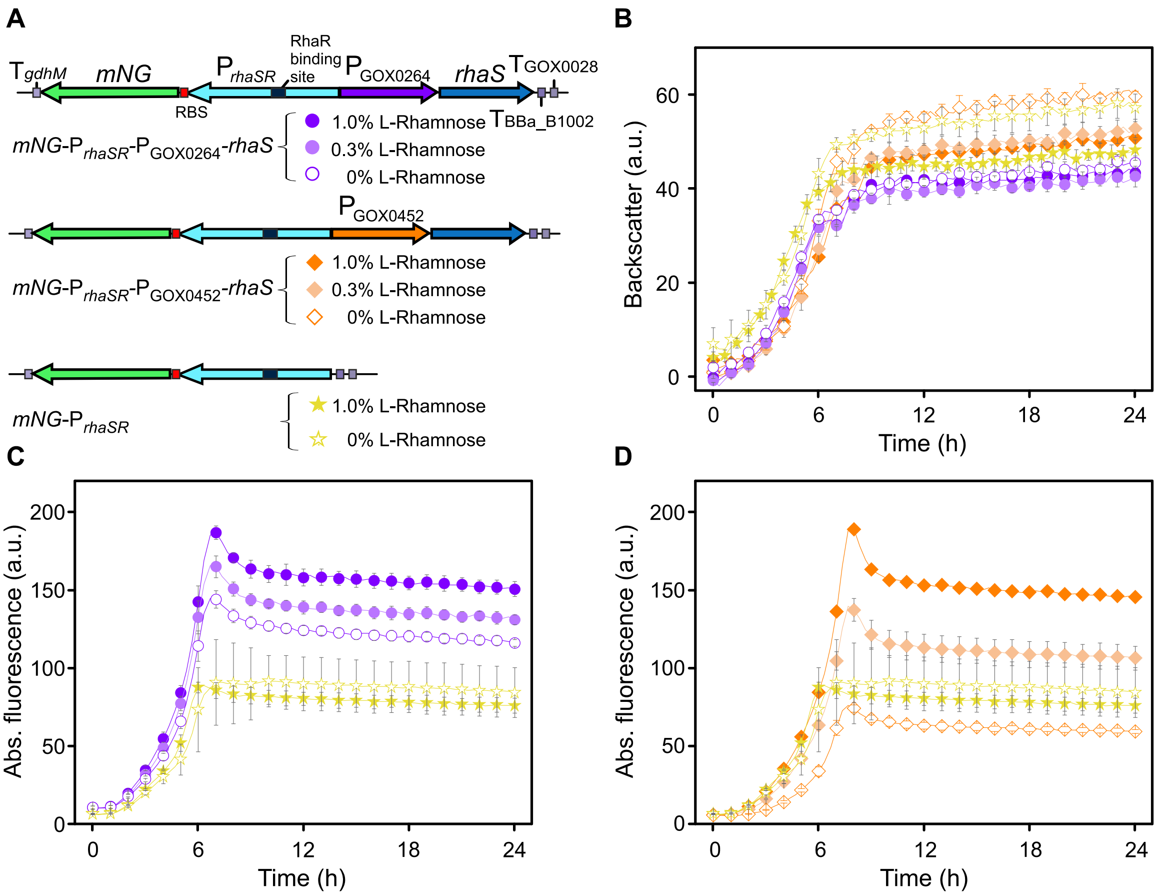

Figure S4 P$_{rhaSR}$-derived *mNG* expression in dependence of *rhaS* expression strength and presence of L-rhamnose.

(A) Schematic illustration of pBBR1MCS-5-derived plasmid variants with insert *mNG*-P$_{rhaSR}$-P$_{GOX0264}$-*rhaS*, *mNG*-P$_{rhaSR}$-P$_{GOX0452}$-*rhaS*, or *mNG*-P$_{rhaSR}$. **(B)** Growth according to backscatter of *G. oxydans* 621H carrying plasmid pBBR1MCS-5-*mNG*-P$_{rhaSR}$-P$_{GOX0264}$-*rhaS*, pBBR1MCS-5-*mNG*-P$_{rhaSR}$-P$_{GOX0452}$-*rhaS*, or pBBR1MCS-5-*mNG*-P$_{rhaSR}$ in microscale BioLector cultivations. Absolute fluorescence of *G. oxydans* 621H with **(C)** plasmid pBBR1MCS-5-*mNG*-P$_{rhaSR}$-P$_{GOX0264}$-*rhaS* and with **(D)** pBBR1MCS-5-*mNG*-P$_{rhaSR}$-P$_{GOX0452}$-*rhaS*, both graphs with the same data from *G. oxydans* 621H carrying pBBR1MCS-5-*mNG*-P$_{rhaSR}$ lacking *rhaS*. L-Rhamnose was supplemented with 0.3% or 1% (w/v). Data represent mean values and standard deviation from two biological replicates with two and three technical replicates each. BioLector settings: backscatter gain 20, fluorescence gain 70.

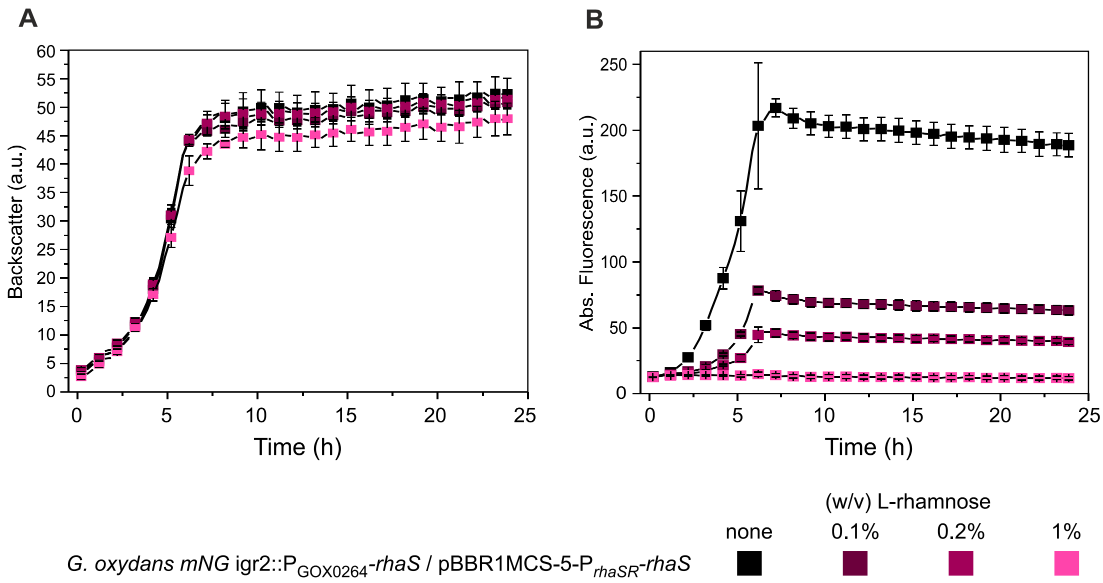

Figure S5 Tunable repression of genomic single-copy P$_{rhaBAD(+RhaS\text{-}BS)}$ by lower L-rhamnose concentrations and plasmid-based multi-copy *rhaS* expression.

(A) Backscatter and **(B)** absolute mNG fluorescence of *G. oxydans* strain *mNG* igr2::P$_{GOX0264}$-*rhaS* with plasmid pBBR1MCS-5-P$_{rhaSR}$-*rhaS* in microscale BioLector cultivations. The strain was cultivated in complex medium with 4% (w/v) D-mannitol. L-Rhamnose was supplemented as indicated. Data represent mean values and standard deviation from two biological replicates (clones) with three technical replicates each. BioLector settings: backscatter gain 20, fluorescence gain 70.

```
G.o.621H  28   AAVKRLIAKGRERGHITFDELNAVLPQDQMSSEQIEDVMAALSEMGIQVIENEDQDEAEA  87
               + +K L+ +G+E+G++T+ E+N  LP+D + S+QIED++  +++MGIQV+E      +
E.c.K12    7   SQLKLLVTRGKEQGYLTYAEVNDHLPEDIVDSDQIEDIIQMINDMGIQVMEEAPDADDLM  66

G.o.621H  88   PAEKEAEGDGEEAEGPQGGNVDAEAASRTDDPVRMYLREMGSVELLSREGEIAIAKRIEA  147
                AE  A+ D  EA       +V++E   RT DPVRMY+REMG+VELL+REGEI IAKRIE
E.c.K12   67   LAENTADEDAAEAAAQVLSSVESEIG-RTTDPVRMYMREMGTVELLTREGEIDIAKRIED  125

G.o.621H 148   GRDEMIGGLCESPLTIRAIISWHERLKDGEMLLRDIVDLEASQGGGPDPEAVEGEEGDEA  207
               G +++    + E P  I  ++  ++R++  E  L D++        G  DP A
E.c.K12  126   GINQVQCSVAEYPEAITYLLEQYDRVEAEEARLSDLI------TGFVDPNA---------  170

G.o.621H 208   SEEDDTAEDENEEGEGDQQEGSGLSLSALEEKLKPEILARFEAIEP-----LYHKLR-KL  261
                 E+D A       G     QE          EE     +       +I+P       + +LR +
E.c.K12  171   --EEDLAPTATHVGSELSQEDLDDDEDEDEEDGDDDSADDDNSIDPELAREKFAELRAQY  228

G.o.621H 262   QIKRIEALTGGEDHSDKSEQTYEKLRHELVSLVEQVHLHNNRIEELVAQIKMQVQKLNNV  321
                + R       G  H+   E+  +      L  + +Q  L    + + LV  +++ + ++
E.c.K12  229   VVTRDTIKAKGRSHATAQEEILK-----LSEVFKQFRLVPKQFDYLVNSMRVMMDRVRTQ  283

G.o.621H 322   EGRMMRLA-ESCKISRDDFLIKYRSRELDPTWLDSISALPGKGWKNLTTKHMDQLRNLRG  380
               E  +M+L  E CK+ + +F+  +    E   TW ++  A+  K W          +++
E.c.K12  284   ERLIMKLCVEQCKMPKKNFITLFTGNETSDTWFNAAIAM-NKPWSEKLHDVSEEVHRALQ  342

G.o.621H 381   EIAALSHETGLPVGEFRRVYATISRGERDSTRAKKEMIEANLRLVISIAKKYTNRGLQFL  440
               ++  +  ETGL + + + +   +S GE  + RAKKEM+EANLRLVISIAKKYTNRGLQFL
E.c.K12  343   KLQQIEEETGLTIEQVKDINRRMSIGEAKARRAKKEMVEANLRLVISIAKKYTNRGLQFL  402

G.o.621H 441   DLIQEGNIGLMKAVDKFEYRRGYKFSTYATWWIRQAITRSIADQAKTIRIPVHMIETINK  500
               DLIQEGNIGLMKAVDKFEYRRGYKFSTYATWWIRQAITRSIADQA+TIRIPVHMIETINK
E.c.K12  403   DLIQEGNIGLMKAVDKFEYRRGYKFSTYATWWIRQAITRSIADQARTIRIPVHMIETINK  462
                                                              -10
G.o.621H 501   LVRTSRQMLHEIGREPAPEELAEKLGMPLEKVRKVLKIAKEPISLETPIGDEEDSHLGDF  560
               L R SRQML E+GREP PEELAE++ MP +K+RKVLKIAKEPIS+ETPIGD+EDSHLGDF
E.c.K12  463   LNRISRQMLQEMGREPTPEELAERMLMPEDKIRKVLKIAKEPISMETPIGDDEDSHLGDF  522

G.o.621H 561   IEDKTAVIPLDAAIQTNLREATTRVLASLTPREERVLRMRFGIGMNTDHTLEEVGQQFNV  620
               IED T   +PLD+A    +LR AT   VLA LT RE +VLRMRFGI MNTD+TLEEVG+QF+V
E.c.K12  523   IEDTTLELPLDSATTESLRAATHDVLAGLTAREAKVLRMRFGIDMNTDYTLEEVGKQFDV  582

G.o.621H 621   TRERIRQIEAKALRKLKHPSRSRKLRSFLDD  651
               TRERIRQIEAKALRKL+HPSRS  LRSFLDD
E.c.K12  583   TRERIRQIEAKALRKLRHPSRSEVLRSFLDD  613
                     -35|     |
                        K593  R599
```

Figure S6 Sequence alignment of σ^{70} from *G. oxydans* 621H and *E. coli* K12.

Regions containing amino acid residues suggested to be involved in –10 and –35 promoter element recognition are marked in blue (Kelly et al., 2018). Amino acid residues K593 and R599 interacting with RhaS in *E. coli* are highlighted in red (Bhende and Egan, 2000;Wickstrum and Egan, 2004).

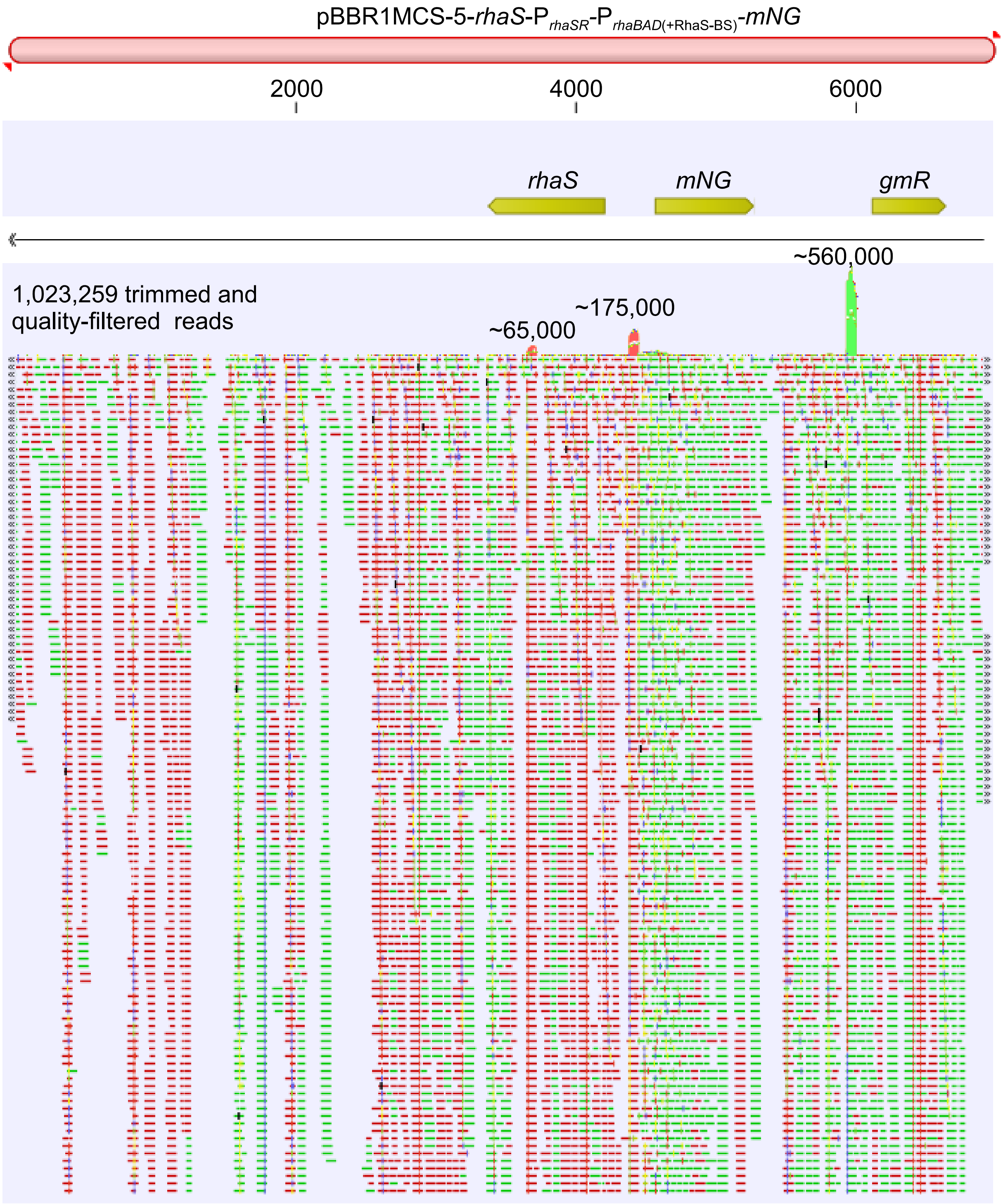

Figure S7 Reads mapping overview for pBBR1MCS-5-*rhaS*-P_{rhaSR}-$P_{rhaBAD(+RhaS\text{-}BS)}$-*mNG*.

Illustration of the overall read mapping shows the three highest reads stacks indicating the three most abundant transcriptional starts on the plasmid. Green color indicates single reads mapping to the plus strand (forward). Red color indicates single reads mapping to the minus strand (reverse). The by far highest stack (~560,000 coverage) corresponded to the annotated promoter region of *gmR* conferring gentamycin resistance and was oriented forward toward *gmR*. The second-highest stack (~175,000 coverage) was oriented reverse and thus toward *rhaS* on the minus strand and the start position of the stack was upstream from P_{rhaSR} within the P_{rhaBAD} region between the -35 and -10 regions from *E. coli*. The third-highest stack (~65,000 coverage) was oriented reverse within the coding region of *rhaS*.

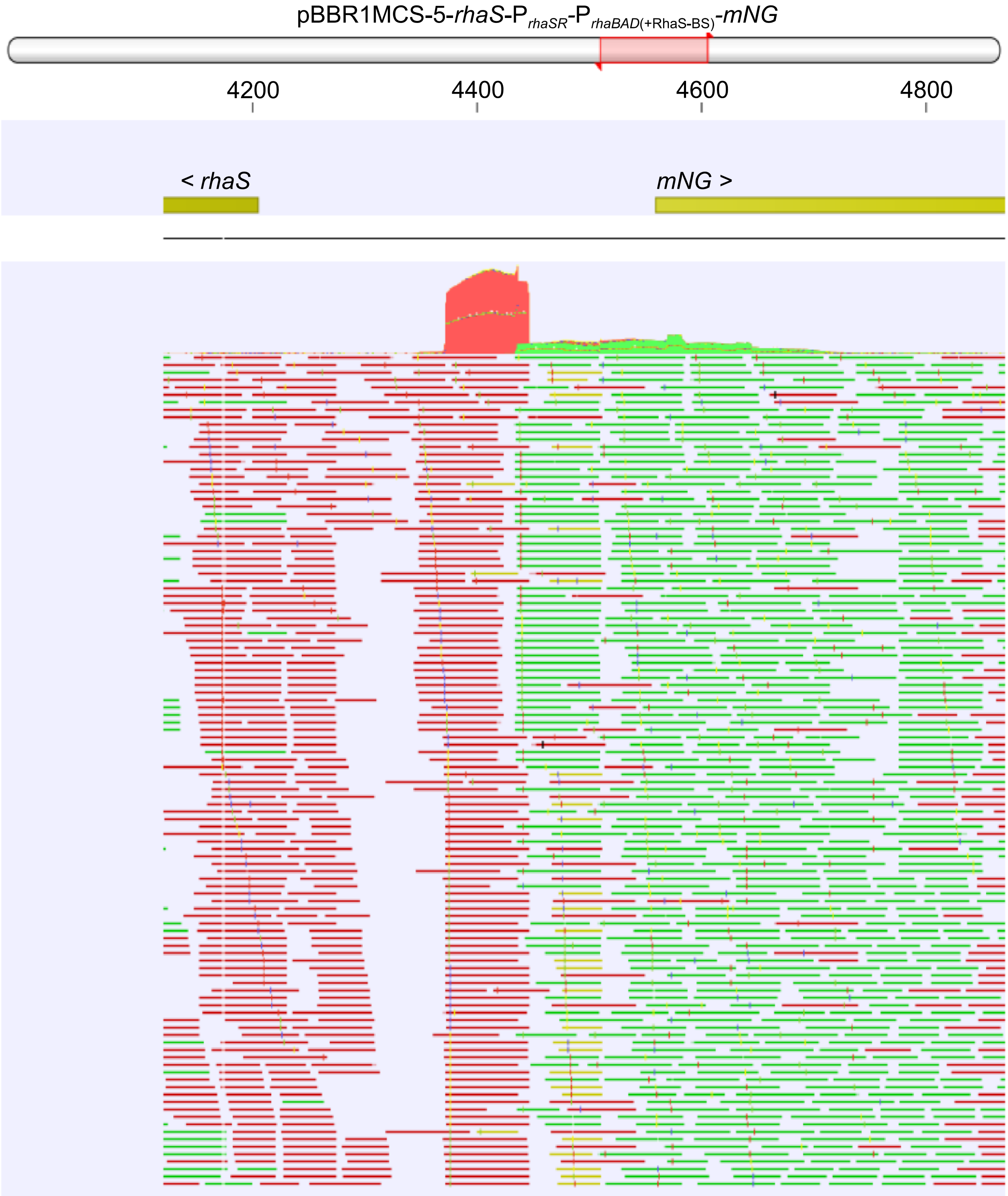

Figure S8 Reads mapping of the P_{rhaSR}-$P_{rhaBAD(+RhaS\text{-}BS)}$ promoter region.

Illustration of the reads mapping shows the potential TSS toward *rhaS* on the minus strand with the start position of the stack upstream from P_{rhaSR} within the P_{rhaBAD} region between the -35 and -10 regions from *E. coli*. For the $P_{rhaBAD(+RhaS\text{-}BS)}$ region and the 5' region of mNG several reads stacks with scattering start positions were found suggesting a multitude of TSSs oriented toward the 3' end of *mNG*. Green color indicates single reads mapping to the plus strand (forward). Red color indicates single reads mapping to the minus strand (reverse).

References

Bhende, P.M., and Egan, S.M. (2000). Genetic evidence that transcription activation by RhaS involves specific amino acid contacts with sigma 70. *J Bacteriol* 182**,** 4959-4969.
Egan, S.M., and Schleif, R.F. (1993). A regulatory cascade in the induction of *rhaBAD*. *J Mol Biol* 234**,** 87-98.
Kelly, C.L., Taylor, G.M., Hitchcock, A., Torres-Mendez, A., and Heap, J.T. (2018). A rhamnose-inducible system for precise and temporal control of gene expression in *Cyanobacteria*. *ACS Synth Biol* 7**,** 1056-1066.
Via, P., Badia, J., Baldoma, L., Obradors, N., and Aguilar, J. (1996). Transcriptional regulation of the *Escherichia coli rhaT* gene. *Microbiology* 142 (Pt 7)**,** 1833-1840.
Wickstrum, J.R., and Egan, S.M. (2004). Amino acid contacts between sigma 70 domain 4 and the transcription activators RhaS and RhaR. *J Bacteriol* 186**,** 6277-6285.
